# Identification of Novel Metabolic Signatures on Human Gut Microbiota: Ellagic Acid, Naringenin, and Phloroglucinol

**DOI:** 10.3390/ijms262211009

**Published:** 2025-11-14

**Authors:** Adriana C. S. Pais, Tânia B. Ribeiro, Ezequiel R. Coscueta, Maria Manuela Pintado, Armando J. D. Silvestre, Sónia A. O. Santos

**Affiliations:** 1 CICECO-Aveiro Institute of Materials, Chemistry Department, University of Aveiro, Camous de Santiago, 3810-193 Aveiro, Portugal; a.c.p.s@ua.pt (A.C.S.P.); armsil@ua.pt (A.J.D.S.); 2 Universidade Católica Portuguesa, CBQF—Centro de Biotecnologia e Química Fina–Laboratório Associado, Escola Superior de Biotecnologia, Rua Diogo Botelho 1327, 4169-005 Porto, Portugal; tribeiro@ucp.pt (T.B.R.); ecoscueta@ucp.pt (E.R.C.); mpintado@ucp.pt (M.M.P.)

**Keywords:** human gut microbiota, phenolic compounds, metabolites, ellagic acid, naringenin, phloroglucinol, fecal fermentation

## Abstract

Phenolic compounds are widely known for their beneficial effects on human health. However, it is essential to understand which low molecular weight metabolites are produced by the gut microbiota, when non-absorbed compounds reach the colon, and whether these metabolites are more biologically active than their precursors. In this context, this study aims to explore the gut microbiota metabolites of relevant phenolic compounds commonly found in the human diet. Therefore, ellagic acid, naringenin, and phloroglucinol were incubated with human feces for 48 h, and the ensuing metabolites were analyzed by ultra-high-performance liquid chromatography with diode array detector coupled to ion trap mass spectrometry (UHPLC-DAD-MS^n^) and gas chromatography–mass spectrometry (GC-MS). Ellagic acid metabolism by the gut microbiota produced a diversity of urolithins, with 8-hydroxyurolithin being identified for the first time. Isomers of 4-hydroxybenzoic, 3,4-dihydroxybenozic, and p-coumaric acids were identified for the first time as naringenin metabolites, while phloroglucinic, 2-hydroxy-3-phenylpropanoic, 3-phenylpropanoic, and 2-phenylacetic acids are reported for the first time as phloroglucinol metabolites. These findings contribute to a more comprehensive understanding of the beneficial health effects of these metabolites through the evaluation of their biological activities in conjunction with their effects on the gut microbiota, thus providing the basis for the development of food supplements, novel probiotics or functional foods.

## 1. Introduction

Currently, the concept “you are what you eat” has become considerably widespread [1]. In fact, there has been a growing awareness that human nutrition is more than just a vital process for the functioning of the human body [2], mainly due to the scientific community’s concerted efforts to elucidate the relationship between the gut microbiota and dietary components [1,3], and particularly bioactive secondary metabolites. The influence exerted by these compounds on the gut microbiota composition has been increasingly studied and reported, and as dietary compounds undergo metabolic transformation by the gut microbiota, a two-way relationship between the gut microbiota and bioactive compounds has been widely recognized by the scientific community [3,4,5,6,7,8,9,10,11,12,13,14].

Dietary phenolic compounds, which are a structurally diverse class of secondary metabolites found in a wide variety of fruits, vegetables, and other plant sources, have been broadly studied due to their vast range of biological activities (including, antioxidant, anti-inflammatory, anti-proliferative, antimicrobial activities, among others) [6,7,8,13,14]. However, ingested phenolic compounds are poorly absorbed in the small intestine (about 10–15%); thus, the remaining bioavailable phenolic compounds reach the colon, where they can be metabolized by the colon microbiota [6,7,14,15]. Therefore, the increasing knowledge about the resultant metabolites and their biological activities, which could be more or less bioactive than their dietary phenolic precursors, represents an important tool in the prevention or control of some diseases [6,13,14,15].

Human gut microbiota is as unique as a fingerprint [8,15,16], comprising about 10^14^ bacterial cells and more than 1000 microbial species [7,8,10,11,15,17] that colonize the human intestinal tract [3,6,8,13,17], and subsequently influence human health [13,14], playing an essential role in the immune system as well as in metabolic functions [3,16,18]. Therefore, the metabolites of phenolic compounds produced are not only dependent on the chemical structure of the precursor phenolic compound [19], but also on the gut microbiota composition [13]. When dietary phenolic compounds reach the colon, they might be enzymatically converted into lower-molecular-weight-phenolic metabolites, which are more bioavailable [11,14]. In fact, gut microbiota bacteria produce specific enzymes (hydrolases, reductases, hydrogenases, decarboxylases, dehydroxylases, among others) that are able to convert phenolic compounds with hydrolysis, cleavage (ring cleavage, demethylation or delactonization), and reduction as the most common phenolic compounds metabolic pathways [3,6,7,8].

The gut microbiota metabolism of some phenolic compounds, namely quercetin [20,21,22,23,24,25,26,27] and catechin [28,29,30,31,32,33,34,35,36,37] derivatives, has already been extensively studied; the bacteria involved and the ensuing metabolites were identified in detail. However, other classes of phenolic compounds have not yet been thoroughly studied. For example, naringenin has been mainly studied using isolated human intestinal bacteria or fermentation with rat gut microbiota bacteria [21,38,39,40,41]. Moreover, some of these studies were performed using complex food matrices as substrates, which could hinder the association between metabolites and respective phenolic compound precursors [42,43,44,45,46,47]. Investigating isolated phenolic compounds and their metabolites is crucial for better understanding the complex interactions occurring within the gut microbiota. When combined with further research on the bioactivities of these metabolites and on how variations in gut bacterial composition influence their metabolism, such studies can provide valuable insights for developing strategies aimed at maximizing the health benefits of phenolic compounds.

Hence, the present study aims to contribute to the identification of metabolites derived from phenolic compounds through human gut microbiota activity during fermentation. Taking into account the results obtained in our previous work on the bioaccessibility and intestinal absorption of some phenolic compounds representative of the most common classes [48], and in light of the findings in the literature regarding the metabolism of phenolic compounds by the gut microbiota as well as their antimicrobial activity or prebiotic effects, ellagic acid, naringenin, and phloroglucinol were submitted to an in vitro batch fermentation with human fecal inoculum, and fermentation samples were further analyzed by UHPLC-DAD-MS^n^ and GC-MS.

## 2. Results and Discussion

### 2.1. Phenolic Compounds Selection

Taking into account the information available in the literature about the metabolism of phenolic compounds by the gut microbiota and our previous work on the bioacessibility and intestinal absorption of phenolic compounds [48], five phenolic compounds were selected for the present study: ellagic acid—a phenolic acid; quercetin—a flavonol; naringenin and naringin—flavanones; and phloroglucinol—a monomeric tannin compound. They ensure representativeness of the most common classes of phenolic compounds.

Gut microbiota is a complex environment with a high diversity of bacteria, and despite the majority belonging to *Bifidobacterium*, *Eubacterium*, *Lactobacillus*, and *Bacteroides*, some pathogens can also be found [49]. Therefore, it was already reported [50] that the antibacterial activity of phenolic compounds (mainly phenolic acids) against *E. coli*, as well as their ability to stimulate the growth of beneficial bacteria (particularly *Lactobacillus* and *Bifidobacterium*), highlights the role of phenolic compounds in human health through the modulation of gut microbiota composition [50]. Before studying how the gut microbiota metabolizes these compounds, their effect on bacterial growth was evaluated to determine whether they have antimicrobial activity or prebiotic effect on pathogenic and probiotic strains, respectively. In this way, the previously selected phenolic compounds were incubated with two probiotic strains (*B. animalis* subsp. *Lactis* BB12 and *L. casei* LC1) for 48 h and with two pathogenic strains (*E. coli* and *S. enterica*) for 24 h (Figures S1–S5, Supplementary Material).

Concerning the effect on probiotic strain growth, ellagic acid, naringenin, and phloroglucinol delayed the death phase of *L*. *casei*. At 48 h, compared with control, the different concentrations of ellagic acid between 12.5 and 200 µg mL^−1^ increased growth by 17.2–12.1%, with no dose-dependent effect, similarly to naringenin and phloroglucinol (50–400 µg mL^−1^), which increased growth by 17.2–15.0% and 16.7–13.4%, respectively. In contrast, both quercetin and naringin accelerated the death phase. Regarding the BB12 growth, the five phenolic compounds showed an inhibitory effect at all tested concentrations, with ellagic acid exhibiting the least pronounced effect (as illustrated in Figure S1a, Supplementary Material).

Overall, after 6 h of incubation, all phenolic compounds promoted an increase in *E. coli* growth compared to controls. However, from 18 to 48 h, all tested concentrations of ellagic acid and naringenin inhibited *E. coli* growth (Figures S1c and S3c, Supplementary Material, respectively).

After 24 h, *S. enterica* growth was inhibited by all the phenolic compounds. Both naringin and phloroglucinol seemed to anticipate the death phase of *S. enterica*, showing at 24 h an inhibition of 28–35% and 52–58% (25–400 µg mL^−1^), respectively.

Higher concentrations (800 and 1600 µg mL^−1^) were tested for all phenolic compounds, except ellagic acid, due to its lower solubility. Predominantly, these concentrations showed a higher inhibitory effect on the growth of all strains. This could be advantageous for pathogenic strains; nevertheless, it is not attractive to *L. casei*. From 18 h, at 800 µg mL^−1^ phloroglucinol and naringin enhanced the growth of *L. casei* when compared to the control.

In this vein, the selection of phenolic compounds and their respective concentrations was based on these results, prioritizing those with minimal negative impact on probiotic strains’ growth while maximizing inhibitory effects on pathogenic ones. Thus, ellagic acid (100 and 200 µg mL^−1^), naringenin (200 and 400 µg mL^−1^), and phloroglucinol (200 and 800 µg mL^−1^) were the ones with the most promising results. A common concentration of 200 µg mL^−1^ was chosen for comparative purposes.

### 2.2. Qualitative and Quantitative Analysis of Phenolic Compounds and Their Metabolites

As mentioned above, this study was carried out to identify the metabolites resulting from the activity of human gut microbiota on different phenolic compounds, particularly ellagic acid, naringenin, and phloroglucinol. There is still limited information regarding naringenin metabolites produced under the dynamics of the human gut microbiota ecosystem, with the literature only reporting the metabolites produced in the presence of isolated human intestinal bacteria or rat gut microbiota [21,38,39,40,41]. Similarly, the metabolites reported for phloroglucinol have so far been studied only using an isolated bacterium [51], making this the first study to identify naringenin and phloroglucinol metabolites under dynamic human gut microbiota. For comparative purposes, ellagic acid was also analyzed under the same conditions as a reference compound, although some of its gut microbiota metabolites have already been reported [52,53,54,55,56].

The phenolic compounds precursors and the resulting metabolites were analyzed by UHPLC-DAD-MS^n^, using the samples at the highest tested concentrations (200, 400, and 800 µg mL^−1^ for ellagic acid, naringenin, and phloroglucinol, respectively), to facilitate detection.

Table S2 (Supplementary Material) summarizes the phenolic compounds precursors and their respective metabolites, including retention time, deprotonated molecular ion [M-H]^−^, the corresponding MS^2^ product ions, as well as the fecal fermentation time in which they were detected. The identification of each compound was performed by comparing its retention time, deprotonated molecular ion [M-H]^−^ and corresponding MS^2^ product ions with those of standards and, when unavailable, with previously published data, as shown in Table S2 (Supplementary Material).

To detect possible low molecular weight metabolites, the in vitro fermentation samples of ellagic acid, naringenin, and phloroglucinol collected at 0 h, 24 h, and 48 h were derivatized and analyzed by GC-MS. In fact, Krumholz et al. [51] reported that phloroglucinol could be converted into lower molecular weight metabolites such as short-chain fatty acids (including butyrate and acetate) [27,51,57]. The analysis of ellagic acid and naringenin fermentation samples by GC-MS did not result in the identification of additional low molecular weight compounds beyond those identified by UHPLC-DAD-MS^n^. Conversely, the GC-MS analysis of phloroglucinol fermentation samples complemented the results obtained through UHPLC-DAD-MS^n^.

The subsequent sections present a detailed discussion of the detection and identification of the in vitro fermentation metabolites of each of the studied compounds obtained by GC-MS or UHPLC-DAD-MS^n^.

#### 2.2.1. Ellagic Acid and Metabolites from In Vitro Batch Fecal Fermentation

**Figure 1:**
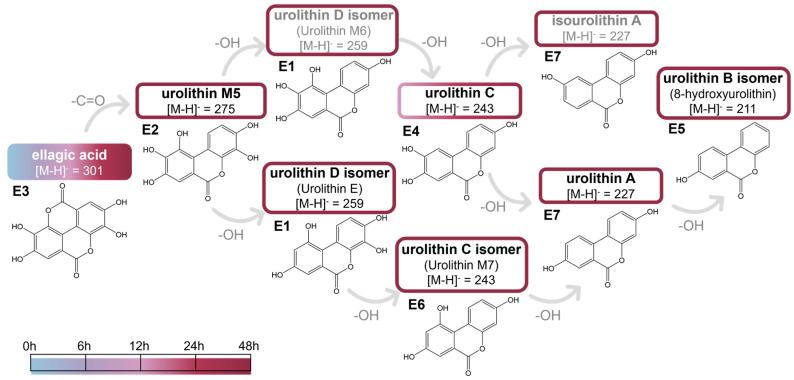
Putative pathway of gut microbiota transformation of ellagic acid, according to the detected metabolites through UHPLC-DAD-MS^2^. The color of the boxes, as indicated by the presented scale, represents the fermentation time at which the compounds were identified.

**Figure 2:**
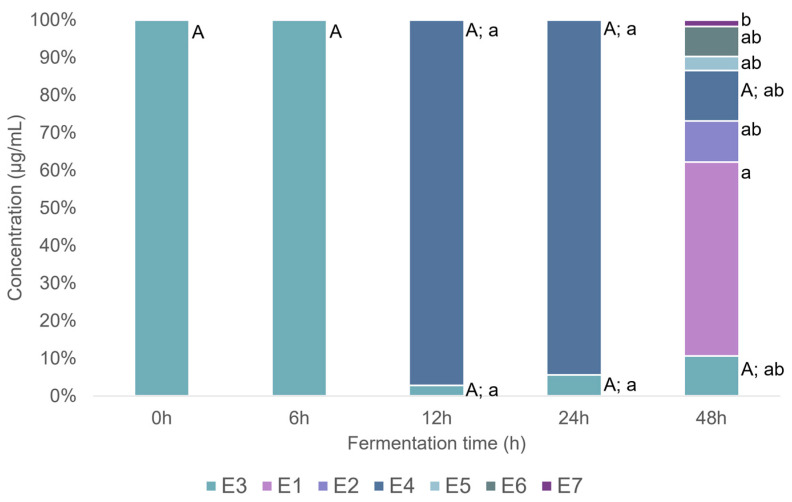
Concentration (µg mL^−1^) of ellagic acid (**E3**) and its metabolites (**E1**—urolithin D isomer, **E2**—urolithin M5, **E4**—urolithin C, **E5**—urolithin B isomer, **E6**—urolithin C isomer, **E7**—(iso)urolithin A) along the fermentation time, obtained through UHPLC-DAD-MS^2^ analysis. Significant differences (*p* < 0.05, Summary Statistical Tables S3–S4) concerning the concentration of each compound at the time of fermentation are indicated by capital letters. Significant differences (*p* < 0.05, Summary Statistical Tables S5–S7) concerning the different phenolic compounds at each time of fermentation are indicated by lowercase letters.

Samples of ellagic acid subjected to in vitro fermentation with human fecal inoculum, and collected after 0, 6, 12, 24, and 48 h were analyzed by UHPLC-DAD-MS^2^, allowing the detection of six metabolites: **E1**, **E2**, **E4**, **E5**, and **E6** (Figure 1 and Figure 2). Significant differences (*p* < 0.05, Summary Statistics Tables S3–S7) concerning the concentration of each compound throughout the fermentation period, as well as among the different phenolic compounds at each time point were evaluated by Kruskal–Wallis test, since these samples did not follow a normal distribution. An exception was observed in the comparison between **E3** and **E4** at 12 h of fermentation, which followed a normal distribution, however homogeneity of variances was not confirmed. Moreover, since at 12 h and 24 h of fermentation only 2 groups (**E3** and **E4**) were considered, the non-parametric Mann–Whitney U test was applied.

Compound **E1** presented a [M-H]^−^ ion at *m*/*z* 259 and product ions at *m*/*z* 241 ([M-H-H_2_O]^−^), 223 ([M-H-2H_2_O]^−^), 215 ([M-H-CO_2_]^−^) and 197 ([M-H-CO_2_-H_2_O]^−^), which are consistent with those of the standard compound urolithin D (Figure 1); however the retention time differed. Therefore, **E1** was identified as an urolithin D isomer. According to the literature data, **E1** could be tentatively identified as urolithin M6 or E [55,58,59,60].

González-Barrio et al. [61] reported that the UV spectra of urolithin derivatives have two characteristic peaks: one between 300 and 380 nm and another one between 240 and 280 nm. These bands may indicate the position of the hydroxy groups, particularly the presence or absence of a hydroxy group at position 9, allowing the identification of isomers based on their UV spectrum [61]. In particular, urolithin M6 (3,8,9,10-tetrahydroxyurolithin) has an absorption at 350 nm, whereas urolithin E (3,4,8,10-tetrahydroxyurolithin) absorbs at about 367 nm [61]. Since the UV spectrum of compound **E1** showed absorption at 274 and 365 nm, this suggests that this tetrahydroxyurolithin lacks a hydroxy group at position 9 [55,61]. Therefore, compound **E1** was tentatively identified as urolithin E, while final confirmation would require authentic standards and/or MS^3^ data.

Similarly, the fragmentation pathways observed for compound **E2** were consistent with urolithin M5 (Figure 1), with its [M-H]^−^ ion at *m*/*z* 275 and its product ions matching the data reported in the literature for this compound [58].

Compound **E3** was identified as ellagic acid, and its retention time, UV spectrum, and MS^2^ fragmentation were consistent with those of the reference standard (Figure 1). Compounds **E4** and **E6** were identified as urolithin C and a corresponding isomer, tentatively assigned as urolithin M7 (Figure 1), based on their [M-H]^−^ ions at *m*/*z* 243. Furthermore, the UV spectrum of compound E6 suggests that this urolithin lacks a hydroxyl group at position 9, as indicated by its absorptions at 279, 353, and 368 nm [59,61], which is consistent with urolithin M7 (3,8,10-trihydroxyurolithin).

Considering the reported data, compound **E7** was identified as (iso)urolithin A (Figure 1) due to its [M-H]^−^ ion at *m*/*z* 227 and its corresponding MS^2^ product ions at *m*/*z* 209 ([M-H-H_2_O]^−^) and 183 ([M-H-CO_2_]^−^) [55,59]. The structural difference between urolithin A and isourolithin A lies in the position of one hydroxy group, located at positions 8 and 9, respectively. Thus, the UV spectrum of compound **E7**, showing peaks at 281, 302, and 358 nm, corresponds to that of urolithin A (3,8-dihydroxyurolithin) [55,59,61].

Finally, compound **E5** showed a [M-H]^−^ ion at *m*/*z* 211 and MS^2^ product ions at *m*/*z* 183 ([M-H-CO]^−^) and 167 ([M-H-CO_2_]^−^), which agree with those of urolithin B, however the retention time did not coincide, and therefore compound **E5** was identified as an urolithin B isomer (Figure 1). According to González-Barrio et al. [61], the UV spectrum of a 9-hydroxyurolithin shows peaks at 256 and 329 nm, which are similar to those of isourolithin A (3,9-dihydroxyurolithin), indicating that the hydroxy at 9-position has a strong influence on the UV spectrum in contrast to the 3-hydroxy group [61]. The UV spectrum of compound **E5** did not match those of urolithin B (3-hydroxyurolithin) (confirmed with a standard reference compound) or isourolithin B (9-hydroxyurolithin). Thus, considering the previous precursor compound (with the closest molecular weight, urolithin A), this compound was tentatively identified as 8-hydroxyurolithin. To the best of our knowledge, this is the first study identifying 8-hydroxyurolithin as a metabolite of ellagic acid obtained by in vitro fecal fermentation, as most studies have reported that (iso)urolithin A or urolithin B are the final metabolites of ellagic acid transformation [52,53,54,55,56,62].

Based on the data above, our results confirm that ellagic acid transformation by gut microbiota occurs via a sequential pathway involving lactone ring cleavage and decarboxylation, followed by successive dehydroxylation steps, as illustrated in Figure 1, in agreement with previously published data [53,63]. According to our findings, and as shown in Figure 2, the first urolithin, **E4** was detected after 12 h of fermentation, at which time most ellagic acid had already been metabolized, while further urolithin metabolites were only detected after 48 h of fermentation. This could be attributed either to the complete conversion of the **E4** precursors (**E1** and **E2**) or to their concentrations being below the limit of detection at earlier fermentation times. These results are not entirely consistent with those of Selma et al. [52,54], who studied ellagic acid metabolism by isolated bacteria and observed that the production of urolithins begins after 2–3 days of incubation.

However, Garcia-Villalba et al. [55] demonstrated that the production of urolithins is influenced by the solvent used to overcome ellagic acid solubility limitations; DMSO, one of the effective solvents in this context, provides significantly different results as it delays urolithin production [55]. Thus, our results (Figure 2) seem to be in accordance with those obtained in the presence of DMSO by García-Villalba et al. [55], in which urolithin A (corresponding to E7) was also found after 42 h of fermentation and the maximum concentration of urolithin M5 (corresponding to **E2**) was detected at 40 h of incubation. The main urolithin produced after 48 h was **E1** (5.1 ± 0.7 µg mL^−1^), which results from the loss of a single hydroxyl group (with a [M-H]^−^ at *m*/*z* 259).

Hence, the metabolism of ellagic acid by the gut microbiota appears to occur through the activity of bacteria able to produce lactonases, decarboxylases, and dehydroxylases, which are responsible for lactone-ring cleavage, decarboxylation, and sequential dehydroxylation, respectively [53,63]. Although urolithin production occurs through sequential dehydroxylation, it seems to depend on gut microbiota composition since there is not always a complete conversion from pentahydroxyurolithin (urolithin M5, **E2**) to dihydroxyuroltihin (urolithin A or isourolithin A, **E7**) or even to hydroxyurolithin (urolithin B, isourolithin B or 8-hydroxyurolithin, **E5**). Three human metabotypes have already been defined for urolithins: metabotype A (urolithin A, **E7** is the final metabolite), metabotype B (besides urolithin A, **E7**, urolithin B and/or isourolithin A, **E7** are found), and metabotype 0 (there is no production of urolithins) [54,56].

As represented in Figure 1, the gut microbiota transformation pathway of ellagic acid may involve a four-step dehydroxylation sequence from urolithin M5, **E2**, (3,4,8,9,10-penta-hydroxyuroltihin) to 8-hydroxyurolithin, **E5**, which was the lowest molecular weight urolithin identified in this study. *Gordonibacter urolithinfaciens* (DSM 27213T) and *G. pamelaeae* (DSM 19378T) are two bacterial species isolated from human feces that have shown the ability to carry out the first two dehydroxylation steps of this pathway, from urolithin M5 (**E2**) to urolithin C (**E4**) through a tetrahydroxyurolithin intermediate [52,64].

Moreover, three possible tetrahydroxyurolithin metabolites were produced and identified through urolithin M5 (**E2**)—urolithin D (3,4,8,9-tetrahydroxyuroltihin), urolithin M6 (3,8,9,10-tetrahydroxyuroltihin), **E1** and urolithin E (3,4,8,10-tetrahydroxyuroltihin), **E1**. Our results suggest that urolithin M5 (**E2**) was transformed into urolithin E, **E1**, consistent with other studies [53,55], where urolithin D was not detected, and urolithin E and M6 (**E1**) were the most common tetrahydroxyurolithins. This may indicate that the hydroxy group at position 10 is more resistant to dehydroxylases activity than those at positions 8 and 9. Indeed, it has been reported that isolated *Gordonibacter* species transform urolithin M5 (**E2**) into urolithin C (**E4**), through urolithin M6, **E1** [52,64]. Additionally, and similarly to the pathway proposed here, Garcia-Villalba et al. [55] showed that urolithin M7, **E6** was the trihydroxyurolithin resulting from the removal of hydroxy group from urolithin E, **E1**.

Furthermore, urolithin A (**E7**), is a dihydroxyuroltihin that can arise from the third dihydroxylation step, either from urolithin C (**E4**) or urolithin M7 (**E6**), in agreement with previously reported data by Gaya et al. [53] and Garcia-Villalba et al. [55]. Three bacterial species capable of performing these third and fourth steps of dehydroxylation, were already isolated, namely *Ellagibacter isourolithinifaciens* CEBA S4A4, from which the final ellagic acid metabolite is isourolithin A, **E7** [54], *Bifidobacterium pseudocatemulatun* INIA P815, which is able to metabolize ellagic acid, **E3**, into urolithin (**E7**) and urolithin B [65], and *Lactococcus garvieae* FUA009, which transforms ellagic acid, **E3**, into a single final metabolite, urolithin A, **E7** [56]. In our study, the lowest molecular weight metabolite detected was 8-hydroxyurolithin, **E5**, based on the UV spectrum of the synthesized reference compound reported by González-Barrio et al. [61]. However, to the best of our knowledge, this is the first study describing 8-hydroxyurolithin, **E5**, as a metabolite of ellagic acid biotransformation by the gut microbiota.

In conclusion, the metabolism of ellagic acid by the gut microbiota produced a diversity of urolithins, which are more readily absorbed in the small intestine than the precursor phenolic compound [55], and are associated with several beneficial effects on human health, as they are known for their anti-inflammatory, antioxidant, antimicrobial activities and their chemopreventive effects against colon cancer [52,63].

#### 2.2.2. Naringenin and Metabolites from In Vitro Batch Fecal Fermentation

**Figure 3:**
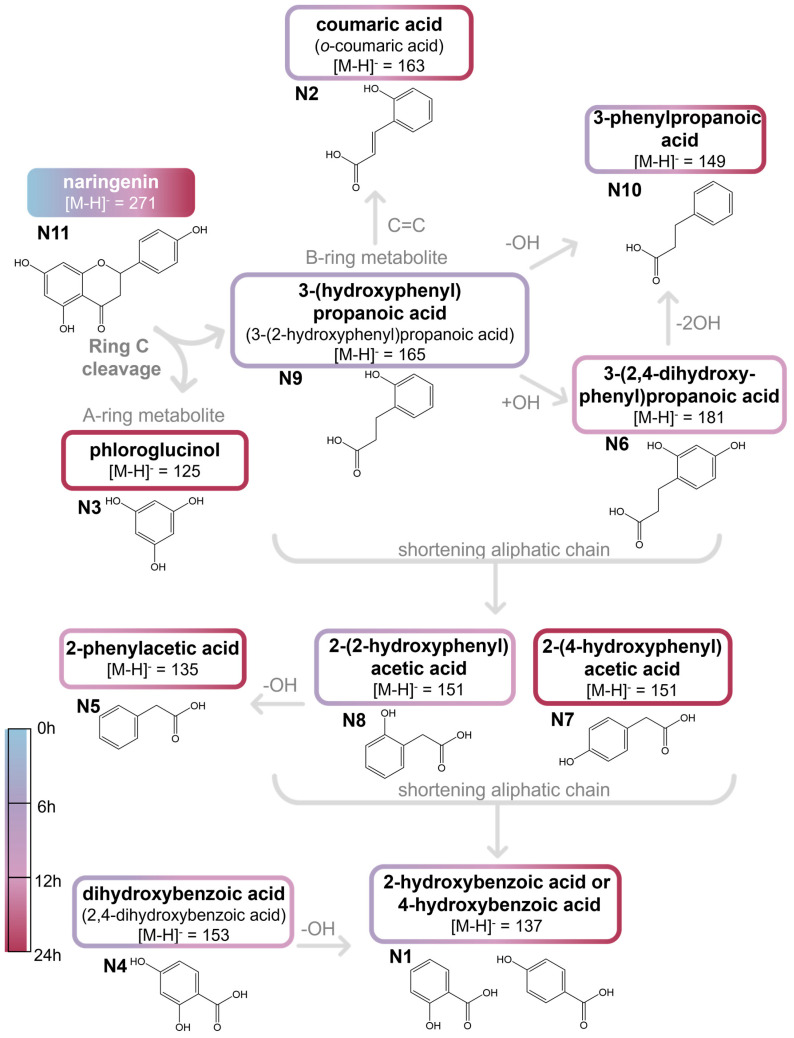
Putative pathway of gut microbiota transformation of naringenin, according to the detected metabolites through UHPLC-DAD-MS^2^. The color of the boxes, as indicated by the presented scale, represents the fermentation time at which the compounds were identified.

**Figure 4:**
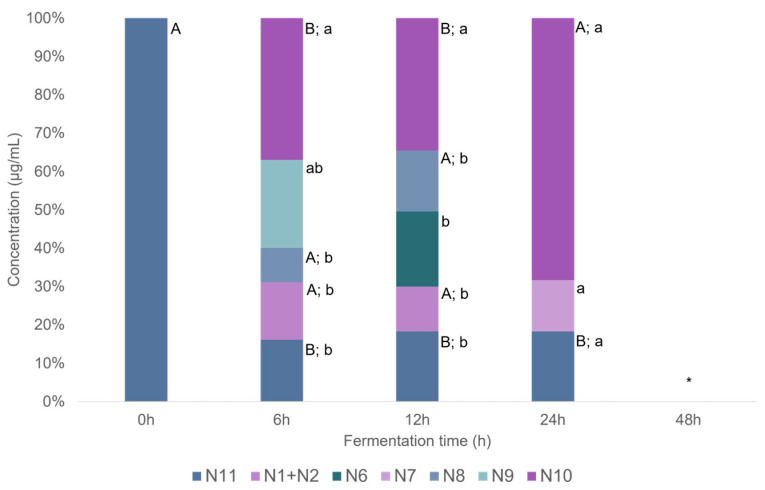
Concentration (µg mL^−1^) of naringenin (**N11**) and the metabolites (**N1**—hydroxybenzoic acid, **N2**—coumaric acid, **N6**—3-(2,4-dihydroxyphenyl)propanoic acid, **N7**—2-(4-hydroxyphenyl)acetic acid, **N8**—2-(2-hydroxyphenyl)acetic acid, **N9**—3-(hydroxyphenyl)propanoic acid, **N10**—3-phenylpropanoic acid) along the fermentation time, obtained through UHPLC-DAD-MS^2^ analysis. Significant differences (*p* < 0.05, Summary Statistical Tables S8–S11) concerning the concentration of each compound along the time of fermentation are indicated by capital letters. Significant differences (*p* < 0.05, Summary Statistical Tables S12–S14) concerning the different phenolic compounds at each time of fermentation are indicated by lowercase letters. * < LOD.

UHPLC-DAD-MS^2^ analysis of naringenin samples after incubation with human feces allowed to detect 10 metabolites, namely **N1**, **N2**, **N3**, **N4**, **N5**, **N6**, **N7**, **N8**, **N9,** and **N10** (Figure 3 and Figure 4). Significant differences (*p* < 0.05, Summary Statistical Tables S8–S14) concerning the concentration of each compound throughout the fermentation period were determined by one-way ANOVA followed by Tukey’s post hoc test, except for **N10** (naringenin) which did not follow a normal distribution and was therefore assessed using the Kruskal–Wallis test. Regarding differences among the phenolic compounds at each fermentation time, only for the samples collected at 24 h, was the Kruskal–Wallis test applied, as those samples did not follow a normal distribution. For the remaining samples, significant differences were evaluated by one-way ANOVA with Tukey’s post hoc test.

Metabolites **N1** and **N2** were identified as hydroxybenzoic acid and coumaric acid isomers, respectively, based on their [M-H]^−^ ions at *m*/*z* 137 and 163, respectively. Further, the MS^2^ fragmentation profile of hydroxybenzoic acid, which shows the loss of carboxyl group, yielding the ion at *m*/*z* 93, did not allow precise identification of the hydroxy group position; therefore, the compound could correspond to either 2-hydroxybenzoic or 4-hydroxybenzoic acid [66] (Figure 3).

Compound **N2** showed MS^2^ product ions at *m*/*z* 135 ([M-H-CO]^−^) and 119 ([M-H-CO_2_]^−^), which matched those of the *p*-coumaric acid standard, although with a different retention time. Thus, compound **N2** was tentatively identified as a *p*-coumaric acid isomer, namely *o*-coumaric acid, following the pathway proposed in Figure 3. Moreover, in the literature, 4-hydroxybenzoic acid has already been reported as a metabolite of naringin (naringenin-7-*O*-rhamnoglucoside) metabolism by the gut microbiota, being first converted into its corresponding aglycone (naringenin) [67].

Compound **N3** was identified as phloroglucinol (Figure 3) based on the comparison of its retention time, UV spectrum, [M-H]^−^ at *m*/*z* 125, and MS^2^ fragmentation profile with those of the standard compound. Similarly, compound **N4** was assigned as a dihydroxybenzoic acid derivative, due to the coincident [M-H]^−^ ion at *m*/*z* 153 and the product ion at *m*/*z* 125 ([M-H-CO]^−^) with those of 3,4-dihydroxybenzoic acid standard compound. However, since the retention time differed, compound **N4** was tentatively identified as 2,4-dihydroxybenzoic acid (Figure 3). Nevertheless, the literature reports only 3,4-dihydroxybenzoic acid as a naringenin metabolite [21].

Compounds **N5** and **N10** were proposed to correspond to 2-phenylacetic and 3-phenylpropanoic acids (Figure 3), respectively, based on their [M-H]^−^ ions (at *m*/*z* 153 and 149, respectively) and their MS^2^ product ions [68,69].

Compound **N6** was identified as 3-(2,4-dihydroxyphenyl)-propanoic acid (Figure 3), with its [M-H]^−^ ion at *m*/*z* 181 and MS^2^ product ion at *m*/*z* 137, corresponding to the loss of a carboxyl group, consistent with the authentic standard compound.

The observed fragmentation pathways, UV spectra and retention times of compounds **N7** and **N8**, were consistent with those of 2-(4-hydroxyphenyl)acetic and 2-(2-hydroxyphenyl)-acetic acids standards, respectively (Figure 3).

Compound **N9** was assigned to 3-(hydroxyphenyl)propanoic acid (Figure 3), based on its [M-H]^−^ ion at *m*/*z* 165 and MS^2^ product ion at *m*/*z* 147 ([M-H-H_2_O]^−^). Since its retention time did not match that of 3-(4-hydroxyphenyl)propanoic acid, the hydroxy group is likely positioned differently, probably corresponding to 3-(2/3-hydroxyphenyl)propanoic acid.

Finally, compound **N11** corresponds to the precursor naringenin (Figure 3), which was identified based on its retention time, UV spectrum, [M-H]^−^ at *m*/*z* 271 and its characteristic product ions, matching those of the standard compound.

Considering the pathway proposed in Figure 3, the first step in naringenin transformation by the gut microbiota appears to be the C-ring cleavage, as **N9** was detected after 6 h of fermentation (Figure 4). This finding is consistent with the transformation observed by other authors [21,70]. Phloroglucinol, also detected in the present study (although below the limit of quantification), has been proposed as the complementary metabolite of this C-ring cleavage. Further, **N10** was detected after 6 h of fermentation and persistent throughout the fermentation process, reaching its maximum concentration at 24 h (2.0 ± 0.14 µg mL^−1^). In fact, 3-(hydroxyphenyl) propanoic acid (**N9**) and 2-(hydroxyphenyl)propanoic acid (**N7** and **N8**) are two of the main products of naringenin metabolism, as reported by Serra et al. [21]. Naringenin transformation by the human gut microbiota appears to occur primarily within the first 24 h of fermentation. This was also observed when naringin was incubated with human faces being first converted to naringenin and subsequently to lower molecular weight metabolites, within the first 24 h of fermentation [71].

The gut microbiota metabolism of naringenin has been proposed to occur through a four-step pathway [12,38]. Initially, the C-ring of naringenin is cleaved, yielding naringenin chalcone via chalcone isomerase, which is subsequently reduced to the dihydrochalcone phloretin by an enoate reductase. These enzymes have been previously purified from *Eubacterium ramulus* and *Clostridium orbiscindens* [38,40]. However, these two metabolites were not detected in the present study, possibly due to their rapid transformation into downstream metabolites, remaining below the detection limit. Following these two first reported steps of naringenin metabolism, two main groups of metabolites are formed, derived from the A- and B-rings after C-ring cleavage (Figure 3) [12,21,70]. At this stage, phloretin is converted by phloretin hydrolase (already isolated from *E. ramulus*) into 3-(4-hydroxyphenyl)propanoic acid and phloroglucinol (**N3**) originating from the B- and A-rings, respectively [41,70]. *C. orbiscindens* is another bacterial strain capable of generating both A-ring and B-ring metabolites [40].

3-(4-Hydroxyphenyl)propanoic acid has been described as the major gut microbiota metabolite of naringenin [21,70]. However, the compound detected in the present study appears to be an isomer of this metabolite, with the hydroxy group located at a different position. Although this metabolite has not been reported previously, two possible pathways could account for this transformation. The first one involves the isomerization of 3-(4-hydroxyphenyl)propanoic acid, derived from the B-ring fragment of naringenin, resulting in a hydroxy group positioned, for example, at positions 2 or 3. An alternative pathway involves the dehydroxylation of 3-(2,4-dihydroxyphenyl)propanoic acid, **N6**, one of the remaining metabolites identified in this study and previously reported as a naringenin metabolite [21]. However, since 3-(2,4-dihydroxyphenyl)propanoic acid was detected at 12 h of fermentation and 3-(hydroxyphenyl)-propanoic acid (**N9**) appeared at 6 h, this hypothesis seems unlikely.

As illustrated in Figure 3, the subsequent successive transformations of hydroxyphenylpropanoic acid occurred through dehydroxylation and β-oxidation, resulting in the formation of phenolic acids with shorter aliphatic side acids. These include hydroxyphenylacetic (**N7** and **N8**) and hydroxybenzoic acids (**N1**), as previously reported [14,21,72].

#### 2.2.3. Phloroglucinol and Metabolites from In Vitro Batch Fecal Fermentation

The UHPLC-DAD-MS^2^ analysis of phloroglucinol after gut microbiota fermentation enabled the identification of two resulting metabolites: compounds **P2** and **P3**. Significant differences (*p* < 0.05, Summary Statistical Tables S15–S21) concerning the concentration of each compound throughout the fermentation period as well as among the different phenolic compounds at each time point, were evaluated by the Kruskal–Wallis test. The homogeneity of variance for **P1** and **P2** was not verified across samples during fermentation and the data for the different phenolic compounds at each time did not follow a normal distribution. Only for the determination of significant differences in P3 during the fermentation time, the one-way ANOVA with Tukey’s post hoc test was used.

**Figure 5:**
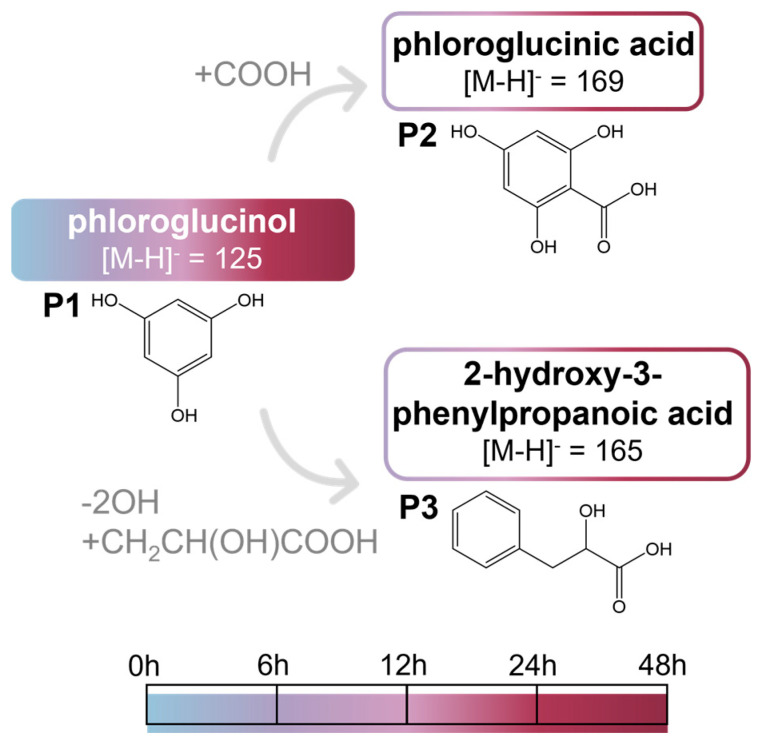
Putative pathway of gut microbiota transformation of phloroglucinol, according to the detected metabolites through UHPLC-DAD-MS^2^. The color of the boxes, as indicated by the presented scale, represents the fermentation time at which the compounds were identified.

Compound **P1** was identified as phloroglucinol (Figure 5) based on its retention time, UV spectrum, [M-H]^−^ at *m*/*z* 125, and the respective product ions, which were coincident with those of the standard compound.

To the best of our knowledge, this is the first study reporting these compounds as gut microbiota metabolites resulting from phloroglucinol transformation. Compound **P2** was identified as phloroglucinic acid (Figure 5) based on its [M-H]^−^ ion at *m*/*z* 169 and its MS^2^ product ions at *m*/*z* 151 ([M-H-H_2_O]^−^), 141 ([M-H-CO]^−^), 125 ([M-H-CO_2_]^−^). Compound **P3** was assigned as 2-hydroxy-3-phenylpropanoic acid (Figure 5) based on its [M-H]^−^ ion at *m*/*z* 165, which upon fragmentation yielded product ions at *m*/*z* 147 ([M-H-H_2_O]^−^) and 121 ([M-H-CO_2_]^−^).

Furthermore, complementing the UHPLC-DAD-MS^2^ results, the GC-MS analysis of phloroglucinol samples collected at 0, 24, and 48 h of fermentation, allowed the identification of four metabolites resulting from phloroglucinol transformation by gut microbiota, as shown in the chromatograms and mass spectra in Figure S6 (Supplementary Material). In addition to compounds **P2** (phloroglucinic acid) and **P3** (2-hydroxy-3-phenylpropanoic acid), both detected in 24 and 48 h samples, the GC-MS analysis also allowed to identify 3-phenylpropanoic acid and 2-phenylacetic acid, both detected in samples after 48 h of fermentation.

**Figure 6:**
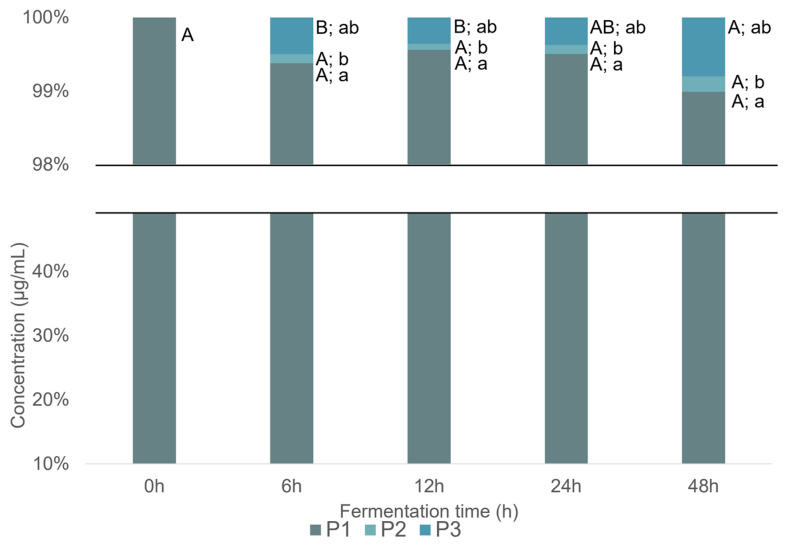
Concentration (µg mL^−1^) of phloroglucinol (**P1**) and the metabolites (**P2**—phloroglucinic acid and **P3**—2-hydroxy-3-phenylpropanoic acid) along the fermentation time, obtained through UHPLC-DAD-MS^2^ analysis. Significant differences (*p* < 0.05, Summary Statistical Table S15–S17) concerning the concentration of each compound at the time of fermentation are indicated by capital letters. Significant differences (*p* < 0.05, Summary Statistical Tables S18–S21) concerning the different phenolic compounds at each time of fermentation are indicated by lowercase letters.

Phloroglucinol (**P1**) was detected in all samples collected during fermentation (Figure 6), indicating that it was not completely metabolized. Based on the UHPLC-DAD-MS^2^ results, **P2** and **P3** were detected after 6 h of fermentation. The concentration of **P2** did not change significantly over time (0.60 ± 0.06 µg mL^−1^ and 0.70 ± 0.06 µg mL^−1^, at 6 h and 48 h, respectively), whereas **P3** concentration showed a significant increase after 24 h of fermentation (Figure 6), reaching about 4.4 ± 0.53 µg mL^−1^ at 48 h. Despite the formation of these metabolites, the concentration of **P1** during the fermentation showed only minor, statistically insignificant variations (Figure 6). This could be attributed to the initially higher concentration of P1compared to that of the detected metabolites.

As mentioned before, to the best of our knowledge this is the first study describing the metabolites of phloroglucinol by the human gut microbiota. Krumholz et al. [51] showed that *Eubacterium oxidoreducens* (isolated from the rumen) is capable of transforming gallic acid into phloroglucinol, which is subsequently metabolized into 3-hydroxy-5-oxohexanoate. Moreover, the gut microbiota metabolism of phlorotannins, found in some edible seaweeds, has not yet been investigated. So far, only the effects of phlorotannin enriched extracts from *Fucus vesiculosus* and from *Silvetia compressa* on human gut microbiota have been studied [73,74].

Phloroglucinol has been identified as a gut-derived metabolite of several flavonoids, such as naringenin, luteolin, kaempferol, and quercetin, among others, and it appears to be one of the lowest-molecular-weight metabolites, resulting in an A-ring fragment from C-cleavage, as mentioned before [21,39,67,75]. The amount of phloroglucinol produced during such fermentation is often below the limit of detection [21], thereby hindering further investigation of its metabolic fate in the large intestine in the presence of gut microbiota. As a low-molecular-weight compound, phloroglucinol would be expected to be readily absorbed and to reach the circulatory system. However, in this study, it was demonstrated that isolated phloroglucinol undergoes biotransformation by the human gut microbiota.

Phloroglucinic, 2-hydroxy-3-phenylpropanoic, 3-phenylpropanoic, and 2-phenylacetic acids are four phenolic acids belonging to benzoic phenyllactic, phenylpropanoic and phenylacetic acids subclasses, respectively, which are structurally related to gut microbiota metabolites of flavonoids [21,39,67,75].

## 3. Materials and Methods

### 3.1. Materials

The reagents used in this study are listed in Table 1.

### 3.2. Preliminary Screening of Antimicrobial and Prebiotic Effects of Phenolic Compounds

Five phenolic compounds previously studied in terms of bioaccessibility and intestinal absorption (ellagic acid, quercetin, naringenin, naringin, and phloroglucinol) [48] were solubilized in pure DMSO and then 4% (*v*/*v*) DMSO; growth medium solutions were used to evaluate their antimicrobial and/or prebiotic effect [76,77,78]. Mueller Hinton broth (MHB) and MRS media were used for pathogenic (*Escherichia coli* and *Salmonella enterica*) and probiotic strains (*Bifidobacterium animalis* subsp. *lactis* BB-12 and *Lactobacillus casei* LC1), respectively. Thus, 200 μL of each 4% (*v*/*v*) DMSO phenolic compound solution, previously filtered (0.22 μm Ø sized), was added in each well of 96-well microplate, to reach a range of final concentrations between 25 and 1600 μg mL^−1^ (except for ellagic acid, which was 12.5–200 μg mL^−1^, due to its low solubility). Then, the inoculum of each bacterial strain was also added (10 μL). As well as two negative (one with only the respective medium and the other with the phenolic compounds solution (4% (*v*/*v*) DMSO), at the higher concentration) and one positive (corresponding to the respective inoculum and medium) controls were performed. For pathogenic bacterial strains, the incubation time was 24 h at 37 °C, whereas for probiotic strains, it was 48 h at 37 °C (under an anaerobic environment using paraffin only to BB-12). Cell growth was monitored by measuring the optical density at 600 nm (OD_600 nm_) of the test culture, against the OD of the bacteria used alone as the control. All experiments were conducted in triplicate.

### 3.3. In Vitro Fermentation Assays

#### 3.3.1. Pool of Human Fecal Inoculum

According to the methodology developed by Carvalho et al. [79], fresh feces from five healthy human donors (2 women and 3 men) were collected into sterile plastic vases, maintaining anaerobic conditions, before being used (maximum of 2 h). Before their participation in the study, all subjects provided informed consent. Prior to collection, the donors were required to demonstrate that they met the following criteria: they were healthy, aged between 18 and 65, had not experienced any food intolerances, and had not taken any probiotic, prebiotic, or antibiotic supplements within the previous six months.

To ensure uniformity and representativeness, similar amounts of each donor fecal sample were pooled together. Subsequently, the fecal inoculum pool was diluted at a concentration of 10% (*w*/*w*) in phosphate-buffered saline (PBS) solution (0.1 M, pH 7.4) and subjected to homogenization in a stomacher for a period of two minutes at a rate of 460 paddle-beats per minute [79].

#### 3.3.2. Fermentation Media

As previously described by Campos et al. [80], the nutrient base medium (NBS) used was constituted by trypticase soya broth (TSB) without dextrose (5.0 g L^−1^), bactopeptone (5.0 g L^−1^), yeast nitrogen base (5.0 g L^−1^), cysteine-HCl (0.5 g L^−1^), 1.0% (*v*/*v*) of salt solution A (100.0 g L^−1^ NH_4_Cl, 10.0 g L^−1^ MgCl_2_·6H_2_O, 10.0 g L^−1^ CaCl_2_·2H_2_O), 0.2% (*v*/*v*) of salt solution B (200.0 g L^−1^ K_2_HPO_4_·3H_2_O), 0.2% (*v*/*v*) resazurin solution (0.5 g L^−1^), and 1% (*v*/*v*) trace minerals (ATCC, 10 mL L^−1^), dissolved in distilled water and the pH adjusted to 6.8. Then, NBS was capped and sterilized by an autoclave.

#### 3.3.3. In Vitro Fermentation

Following sterilization, all components of fecal fermentation were placed in an anaerobic cabinet (5% H_2_, 10% CO_2_, and 85% N_2_). Previously to inoculation, phenolic compounds solutions in DMSO (ellagic acid and naringenin 10 mg mL^−1^ and phloroglucinol 20 mg mL^−1^) were added to tubes with NBS (4% DMSO) to achieve a final concentration of 100 and 200 μg mL^−1^ of ellagic acid, 200 and 400 μg mL^−1^ of naringenin, and 200 and 800 μg mL^−1^ of phloroglucinol. Then, each tube was inoculated with fecal slurry (final concentration of 2% (*v*/*v*)) and incubated at 37 °C for 48 h without agitation [79,80]. Negative (only fecal inoculum) and positive (with a well-known prebiotic, fructooligosaccharides (FOS) controls were also carried out, as well as a respective control of each phenolic compound at 200 μg mL^−1^ with inoculum. To ensure that fermentation assay conditions were not perturbed or interrupted, individual tubes were prepared for each compound, allowing sample collection at 0, 6, 12, 24, and 48 h of fermentation and immediately centrifuged at 16,000× *g* for 5 min. Both resulting pellet and supernatants were stored at −80 °C, the pellet conserved for genomic DNA extraction, whereas the supernatants were stored for further identification and quantification of phenolic compounds and resultants metabolites analysis. All the experiments were conducted in triplicate.

### 3.4. Analysis of Phenolic Compounds and Their Metabolites

#### 3.4.1. Qualitative and Quantitative UHPLC-DAD-MS^n^ Analysis

Fermentation samples were freeze-dried, solubilized in methanol and filtered through a 0.2 μm PTFE syringe filter before injection (20 µL) in a UHPLC system equipped with a variable loop Accela autosampler (set at 16 °C), an Accela 600 LC pump, and an Accela 80 Hz photodiode array detector (DAD) (Thermo Fisher Scientific, San Jose, CA, USA). The column used for separation was a Hypersil Gold C_18_ (100 mm × 2.1 mm × 1.9 μm) column protected with a pre-column (10 mm × 2.1 mm × 1.9 μm), both supplied by ThermoFisher (Thermo Fisher Scientific, San Jose, CA, USA). The mobile phase was composed of acetonitrile (A) and water:acetonitrile (99:1, *v*/*v*) (B), each containing 0.1% (*v*/*v*) of formic acid. The gradient elution was carried out with a flow rate of 0.4 mL min^−1^, at 40 °C, for 29 min, as follows: 0–1 min: 99% B; 1–17 min: 99–73% B, 17–20 min: 73–0% B, 20–25 min: 0–99% B, followed by re-equilibration of the column at 99% B for 4 min. The 200–600 nm UV spectra were recorded, and chromatograms were also obtained at 270, 285 and 370 nm.

An LCQ Fleet ion trap mass spectrometer (ThermoFinnigan, San Jose, CA, USA) was coupled to the UHPLC system with an electrospray ionization (ESI) source, operating in negative mode, as previously described by Santos et al. [81]. The analysis was carried out with the following conditions: spray voltage of 5 kV; capillary temperature 360 °C; capillary and tune lens voltages at −28 V and −115 V, respectively; CID-MS^n^ experiments in the range of *m*/*z* 100–1500; collision energy from 15 and 45 (arbitrary units), using helium as collision gas. The data acquisition was carried out by using Xcalibur^®^ data system 4.0.27.19 (ThermoFiningan, San Jose, CA, USA).

Ellagic acid, naringenin, phloroglucinol, and several other standard compounds, representative of identified metabolites, were solubilized in HPLC-grade methanol with at least five concentrations to obtain standard curves. The quantification of each phenolic compound and identified metabolites was carried out through the corresponding linear regression equation or that of the most similar standard compound (Table S1, Supplementary Material).

#### 3.4.2. Qualitative GC-MS Analysis

Fermentation samples were filtered through a 0.2 μm nylon syringe filter before being freeze-dried. Before GC-MS analysis, these dried samples were dissolved in 50 µL pyridine. Further, 250 µL of *N,O*-bis(trimethylsilyl)trifluoro-acetamide, and 50 µL of trimethylchlorosilane were added, standing at 70 °C for 30 min, in order to convert the compounds containing hydroxyl and carboxyl groups into trimethylsilyl (TMS) ethers and esters, respectively [82,83].

After derivatization, the samples were analyzed by GC-MS following previously described methodologies [83] on a GC-MS-QP2020 NX (Shimadzu, Columbia, MD, USA) equipped with a DB-5 J&W capillary column (30 m × 0.32 mm inner diameter, 0.25 µm film thickness). The carrier gas used was helium (40 cm s^−1^). The chromatographic conditions were as follows: initial temperature, 80 °C for 5 min; temperature gradient, 4 °C min^−1^; final temperature, 260 °C; temperature gradient 2 °C min^−1^; final temperature, 285 °C for 8 min; injector temperature, 250 °C; transfer-line temperature, 290 °C; and split ratio, 1:50. The mass spectrometer was operated in the electron impact mode with an energy of 70 eV, and data were collected at a rate of 1 scan s^−1^ over a range of *m*/*z* 33–700. The ion source was kept at 250 °C [83]. The data acquisition was carried out by using GC Solution 4.20 (Shimadzu, Columbia, MD, USA).

The identification of TMS derivatives was carried out by comparing their mass spectra with the GC-MS spectral library (NIST14) and by comparing their mass spectra with published data or by injection of the available standards.

#### 3.4.3. Statistical Analysis

In the present study, statistical analysis was carried out using OriginPRO 2024 v10.1.0.170. We first checked whether the data followed a normal distribution. In cases where we confirmed a normal distribution, we assessed the homogeneity of variances using Levene’s test (*p*-value > 0.05).

For samples with normal distribution and homogeneous variance, a one-way ANOVA was employed to compare the concentration of each identified phenolic compound over fermentation time and the concentration of the identified compounds at each specific time. To determine a pairwise comparison of means, we applied Tukey’s post hoc test. However, we opted for the non-parametric Kruskal–Wallis test for cases where the data did not follow a normal distribution or when the homogeneity of variances was not confirmed, with the exception of the cases where only two groups were considered in statistical analysis, in which Mann–Whitney test U was applied

The variation in the sample number (*n*) can be attributed to two possible factors: first, the loss of some samples during the process of freeze-drying; and second, a variation in the replicate injections that were performed twice.

## 4. Conclusions

In this study, the gut microbiota metabolic pathways of ellagic acid, naringenin, and phloroglucinol were proposed. Naringenin and phloroglucinol metabolites produced by the gut microbiota, rather than by isolated bacterial strains, were investigated for the first time. An isomeric form of urolithin with a lower molecular weight (urolithin B) was identified for the first time as an ellagic acid metabolite in studies involving human fecal fermentation. Naringenin metabolites were generated through C-ring cleavage, yielding phloroglucinol and 3-(hydroxyphenyl)propanoic acid, which was subsequently metabolized into phenylpropanoic, phenylacetic, and benzoic acids.

The use of two chromatographic techniques (UHPLC-DAD-MS^n^ and GC-MS) provided detailed insights into the phloroglucinol metabolic pathway, which includes phloroglucinic, 2-hydroxy-3-phenylpropanoic, 3-phenylpropanoic, and 2-phenylacetic acids. To the best of our knowledge, this is the first study identifying these metabolites as products of phloroglucinol fermentation by human gut microbiota.

Therefore, this study provides a valuable contribution to the understanding of phenolic compound metabolism by human gut bacteria, particularly regarding the metabolic pathways of naringenin and phloroglucinol. In the absence of a food matrix, the results must be interpreted cautiously to avoid overgeneralized conclusions. Nevertheless, these findings enhance our understanding of the complex interactions between phenolic compounds and gut microbiota. Furthermore, they lay the foundation for future studies focused on the biological activities and potential health benefits of these metabolites, which represent an essential step toward the development of effective phenolic compound-based nutraceuticals and pharmaceuticals.

## Figures and Tables

**Table 1:** List of reagents used in this study.

Reagent Name	Supplier
2-(2-Hydroxyphenyl)acetic acid (99% purity)	Sigma-Aldrich (Madrid, Spain)
2-(4-Hydroxyphenyl)acetic acid (>98% purity)	Sigma-Aldrich (Madrid, Spain)
3-(2,4-Dihydroxyphenyl)propanoic acid (95% purity)	Sigma-Aldrich (Madrid, Spain)
3-(4-Hydroxyphenylpropanoic acid (98% purity)	Sigma-Aldrich (Madrid, Spain)
3,4-Dihydroxybenzoic acid (97% purity)	Thermo-scientific (Slovakia)
Acetonitrile (HPLC grade)	Honeywell (Villepinte, France)
Bile salts (48305)	Sigma-Aldrich (St. Louis, MO, USA)
Calcium chloride (102378)	Merck (New Jersey, USA)
D-(+)-Glucose (G8270)	Sigma-Aldrich (St. Louis, MO, USA)
D-(+)-Maltose monohydrate (63419)	Sigma-Aldrich (St. Louis, MO, USA)
Ellagic acid (96% purity)	Sigma-Aldrich (Madrid, Spain)
Heme chloride (A11165)	Alfa Aesar (Stoughton, MA, USA)
L-Cysteine hydrochloride (1.02839.0100)	Merck (Rahway, NJ, USA)
Lysozyme (P00698)	Sigma-Aldrich (St. Louis, MO, USA)
Magnesium sulfate hexahydrate (459337)	Carlo Erba (Emmendingen, Deutschland)
N,O-bis(trimethylsilyl)trifluoroacetamide (99% purity)	Merck (Madrid, Spain)
Naringenin (98% purity)	Biosynth^®^ CarboSynth (Bratislava, Slovakia)
Naringin (95% purity)	Biosynth^®^ CarboSynth (Bratislava, Slovakia)
NZY Tissue gDNA kit for DNA extraction (MB13502)	NZYTech (Lisbon, Portugal)
*p*-Coumaric acid (≥98% purity)	Sigma-Aldrich (Madrid, Spain)
Phloroglucinol (99% purity)	Sigma-Aldrich (Madrid, Spain)
Potassium dihydrogen phosphate (1.04871.1000)	Merck (Rahway, NJ, USA)
Pyridine (≥ 99.5% purity)	Merck (Madrid, Spain)
Quercetin (95% purity)	Sigma-Aldrich (Madrid, Spain)
Resazurin sodium salt (199303)	Sigma-Aldrich (St. Louis, MO, USA)
Sodium bicarbonate (1.06329.1000)	Merck (Rahway, NJ, USA)
Sodium chloride (31434)	Honeywell (Charlotte, NC, USA)
Solvent Filtration Apparatus 58061	Supelco (Bellefonte, PA, USA)
Soya peptone (84616.0500)	VWR Chemicals (Radnor, PA, USA)
Tetracosane (99% purity)	Merck (Madrid, Spain)
Trimethylchlorosilane (99% purity)	Merck (Madrid, Spain)
Tris-EDTA buffer (TE, 10× concentrate, PPB010)	Sigma-Aldrich (St. Louis, MO, USA)
Tryptone (A1401 HA)	Biokar Diagnostics (Cedex, France)
Tween 80 (28830,291)	VWR Chemicals (Radnor, PA, USA)
Urolithin B (≥98% purity)	Cayman Chemical company (Ann Arbor, MI, USA)
Urolithin C (>97% purity)	Sigma-Aldrich (Madrid, Spain)
Urolithin D (95% purity)	Sigma-Aldrich (Madrid, Spain)
Vitamin K1 (V3501)	Sigma-Aldrich (St. Louis, MO, USA)
Water (HPLC grade)	Merck (Darmstadt, Germany)
Yeast extract (A1202)	Biokar Diagnostics (Paris La Défense Cedex, France)

## Data Availability

The original contributions presented in this study are included in the article/Supplementary Material. Further inquiries can be directed to the corresponding author.

## Supplementary Materials

The supporting information can be downloaded at https://www.mdpi.com/article/10.3390/ijms262211009/s1. References [84,85,86] are cited in the supplementary materials.

## Abbreviations

The following abbreviations are used in this manuscript:

UHPLC-DAD-MSn	Ultra-high performance liquid chromatography with diode array detector coupled to an ion trap mass spectrometer
GC-MS	Gas chromatography coupled to mass spectrometer
MHB	Mueller Hinton broth
TMS	Trimethylsilyl
