# Supplementary material for: Identification of Novel Metabolic Signatures on Human Gut Microbiota: Ellagic Acid, Naringenin, and Phloroglucinol
Source: Int J Mol Sci. 2025 Nov 14;26(22):11009. doi: 10.3390/ijms262211009 (PMC12652937; doi:10.3390/ijms262211009)
Supplement: Supplementary file 1 [file ijms-26-11009-s001.zip › ijms-3893613-supplementary.pdf]

Table S1 Linear regression equations used for UHPLC-UV quantification of phenolic compound and respective metabolites.

Standard compound	λ (nm)[1]	Conc. Rang (μg mL^{-1})	Linear regression equation[2]	R^2	LOD[3] (μg mL^{-1})	LOQ[4] (μg mL^{-1})
phloroglucinol	270	2.04 - 81.60	$y = 10866x + 34673$	0.9965	7.11	21.55
2-(2-hydroxyphenyl) acetic acid	270	0.20 - 10.20	$y = 72648x + 7641.4$	0.9965	0.84	2.56
2-(4-hydroxyphenyl) acetic acid	270	0.52 - 20.80	$y = 56082x + 21402$	0.9996	0.60	1.81
3,4-dihydroxybenzoic acid	270	0.20 - 39.20	$y = 244575x - 73153$	0.9994	1.17	3.56
3-(4-hydroxyphenyl) propanoic acid	270	0.20-40.00	$y = 39321x - 5236.9$	0.9998	0.70	2.12
3-(2,4-dihydroxyphenyl) propanoic acid	270	0.21 - 42.00	$y = 43850x - 2421.1$	0.9992	1.51	4.59
urolithin B	270	0.18 - 18.00	$y = 700564x - 60339$	0.9997	0.54	1.65
urolithin C	270	0.18 - 52.50	$y = 336108x - 187501$	0.9992	1.80	5.45
urolithin D	270	0.20 - 10.00	$y = 426083x - 43178$	0.9996	0.28	0.84
p-coumaric acid	285	0.18 - 4.43	$y = 763136x - 4691.1$	0.9991	0.20	0.60
naringenin	285	0.20 - 4.96	$y = 449428x + 17129$	0.9973	0.38	1.17
ellagic acid	370	0.23 - 11.40	$y = 326638x + 29888$	0.9984	0.64	1.93

[1] Wavelength used in HPLC-UV quantification; [2] y and x represent peak area and concentration in μg mL^{-1}, respectively; [3] LOD, limit of detection; [4] LOQ, limit of quantification.

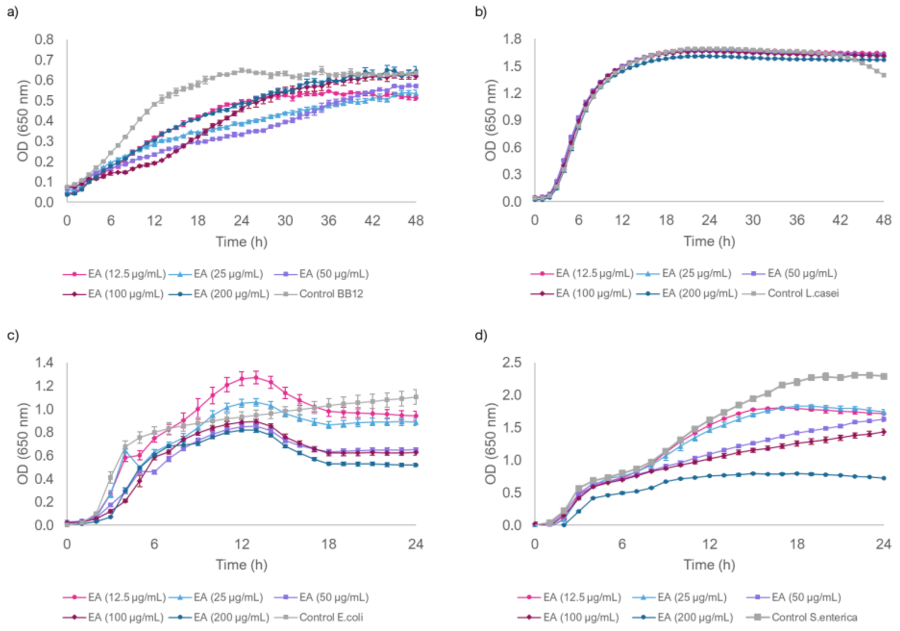

Figure S1 Growth curves (measured by absorbance at 650 nm) of probiotics strains - **a)** BB12, **b)** *L.casei*; and pathogenic strains - **c)** *E.coli* and **d)** *S.enterica* in presence of ellagic acid (EA) at concentrations between 12.5 and 200 µg mL^{-1}.

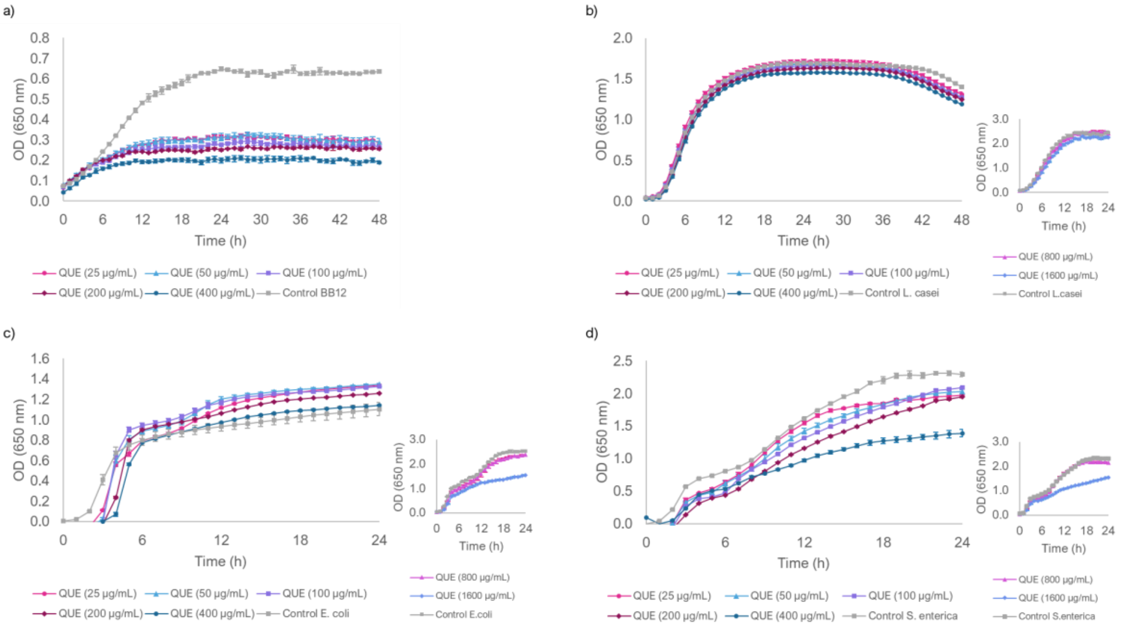

Figure S2 Growth curves (measured by absorbance at 650 nm) of probiotics strains - **a)** BB12, **b)** *L.casei*; and pathogenic strains - **c)** *E.coli* and **d)** *S.enterica* in presence of quercetin (QUE) at concentrations between 25 and 1600 µg mL^{-1}.

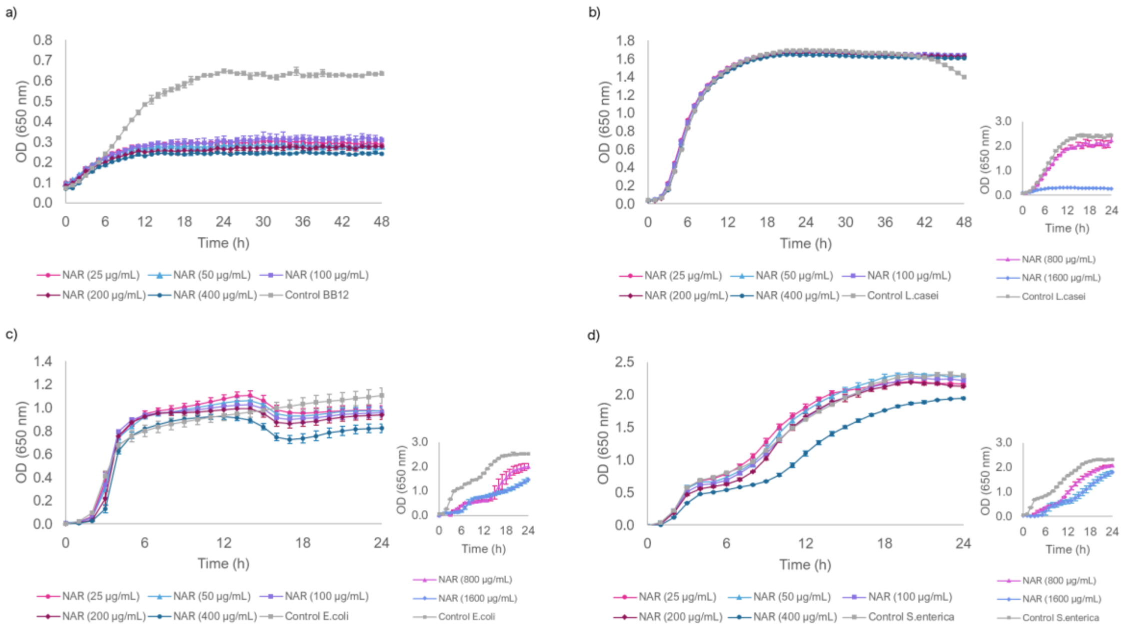

Figure S3 Growth curves (measured by absorbance at 650 nm) of probiotics strains - **a)** BB12, **b)** *L.casei*; and pathogenic strains - **c)** *E.coli* and **d)** *S.enterica* in presence of naringenin (NAR) at concentrations between 25 and 1600 μg mL^{-1}.

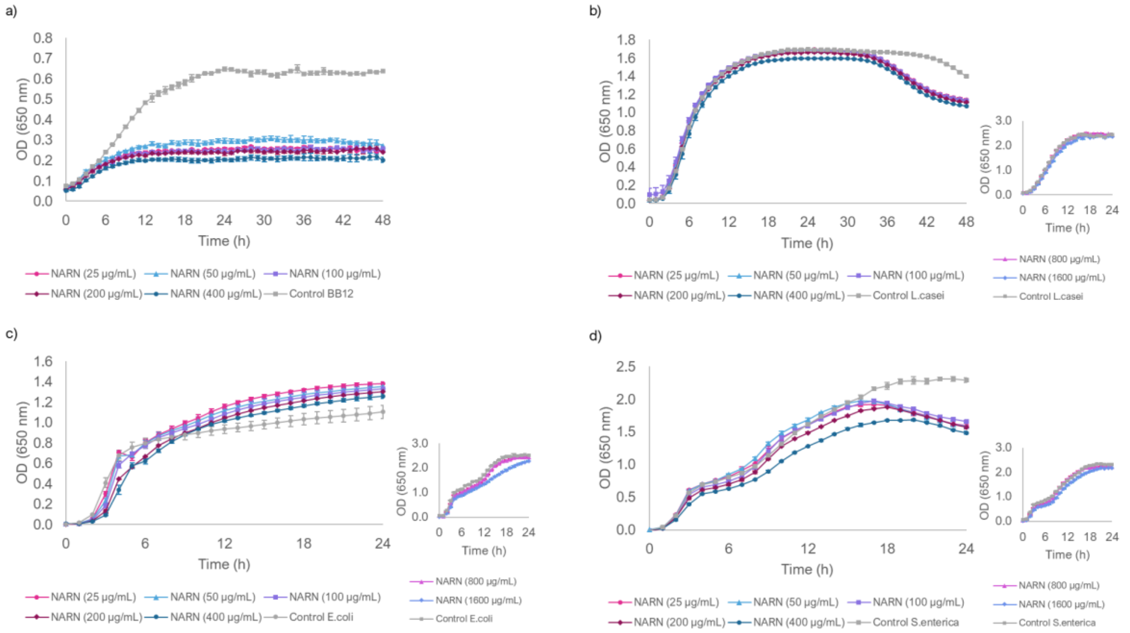

Figure S4 Growth curves (measured by absorbance at 650 nm) of probiotics strains - **a)** BB12, **b)** *L.casei*; and pathogenic strains - **c)** *E.coli* and **d)** *S.enterica* in presence of naringin (NARN) at concentrations between 25 and 1600 μg mL^{-1}.

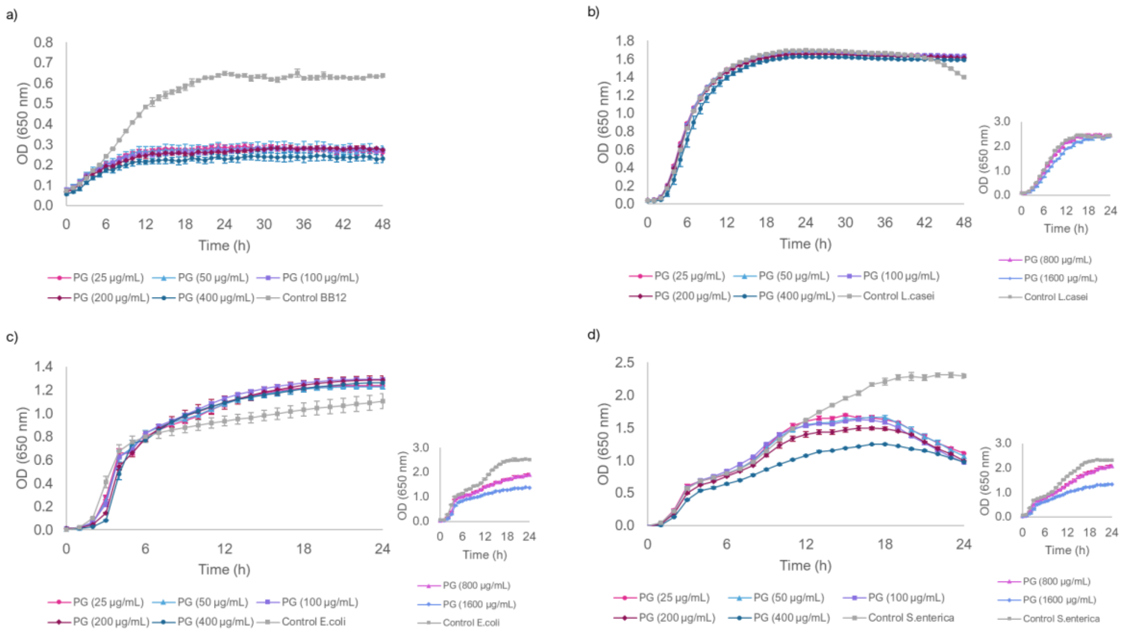

Figure S5 Growth curves (measured by absorbance at 650 nm) of probiotics strains - **a)** BB12, **b)** *L.casei*; and pathogenic strains - **c)** *E.coli* and **d)** *S.enterica* in presence of phloroglucinol (PG) at concentrations between 25 and 1600 µg mL^{-1}.

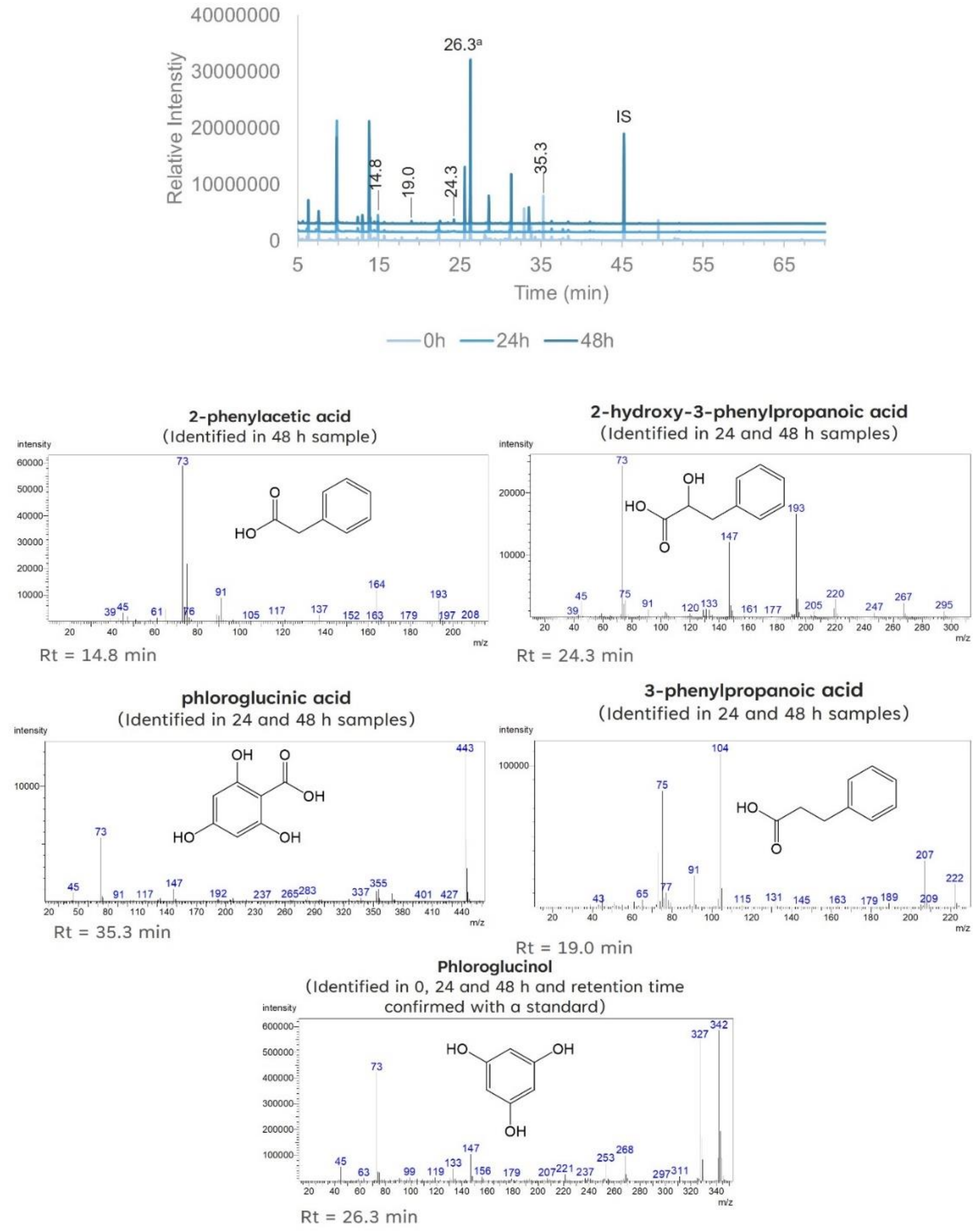

Figure S6 GC-MS chromatograms of phloroglucinol samples collected at 0h, 24h and 48h and respective mass spectra of identified compounds. Phloroglucinol retention time was confirmed through the respective standard compound. (IS – internal standard).

Table S2 Precursors PCs and respective metabolites identified during the *in vitro* fecal fermentation by UHPLC-DAD-MS^n.

No.	Compound	Rt (min)	$[M-H]^-$ (*m/z*)	MS^2 ions (*m/z*)	***In vitro* fecal fermentation time (h)**					Id.
					0	6	12	24	48	
**EA and metabolites**										
**E1**	urolithin D isomer	1.38	259	241, 223, 215, 197, 171				×	×	[1–4]
**E2**	urolithin M5	8.77	275	275, 257, 247, 229					×	[1, 4]
**E3**	**EA**	10.39	301	301, 257, 229	×	×	×	×	×	Co.
**E4**	urolithin C	11.6	243				×	×	×	Co.
**E5**	urolithin B isomer	16.8	211	211, 183, 167					×	[4]
**E6**	urolithin C isomer	20.1	243	243, 225					×	[1, 4–6]
**E7**	(iso)urolithin A	20.4	227	227, 209, 183					×	[1–4, 7]
**NAR and metabolites**										
**N1**	hydroxybenzoic acid[a]	1.38	137	137, 93		×	×	×		[7]
**N2**	coumaric acid[a]	1.38	163	135, 119			×	×		[7]
**N3**	PG	1.55	125	125, 89				×		Co.
**N4**	dihydroxybenzoic acid	2.27	153	153, 125		×	×			[7, 8]
**N5**	2-phenylacetic acid	3.65	135	99, 91			×	×		[9]
**N6**	3-(2,4-dihydroxyphenyl)propanoic acid	6.59	181	137			×			Co.
**N7**	2-(4-hydroxyphenyl)acetic acid	6.59	151	107, 93				×		Co.
**N8**	2-(2-hydroxyphenyl)acetic acid	7.76	151	107		×	×			Co.
**N9**	3-(hydroxyphenyl) propanoic acid	8.12	165	165, 147		×				[10, 11]
**N10**	3-phenylpropanoic acid	9.41	149	149, 105, 93		×	×	×		[11]
**N11**	**NAR**	17.5	271	227, 177, 151, 107, 93, 83	×	×	×	×		Co.
**PG and metabolites**										
**P1**	**PG**	1.50	125	125, 89, 79, 57	×	×	×	×	×	Co.
**P2**	phloroglucinic acid	2.90	169	151, 141, 125		×	×	×	×	
**P3**	2-hydroxy-3-phenyl propanoic acid	9.04	165	147, 121		×	×	×	×	

Co. – Co-injection of the authentic standard. [a] Co-eluted

Table S3 Statistical analysis of E3 (ellagic acid) concentrations along fermentation time.

**Normality test**			**Non-parametric test**						**Post-hoc test**				
**Shapiro-wilk test**			**Kruskal-wallis test**						**Dunn's Test**				
df	stat	*p*-value		*n*	Mean Rank	Chi-Square	*df*	Prob>Chi-Square		Mean Rank Diff	Z	Prob	Sig
14	0.669	1.786E-4	**0h**	3	10	10.990	4	0.027	**0h 6h**	4.667	1.366	0.999	NS
			**6h**	3	5.333				**0h 12h**	7.5	1.964	0.495	NS
			**12h**	2	2.5				**0h 24h**	5	1.464	0.999	NS
			**24h**	3	5				**0h 48h**	-3	-0.878	0.999	NS
			**48h**	3	13				**6h 12h**	2.833	0.742	0.999	NS
									**6h 24h**	0.333	0.098	0.999	NS
									**6h 48h**	-7.667	-2.245	0.248	NS
									**12h 24h**	-2.5	-0.655	0.999	NS
									**12h 48h**	-10.5	-2.750	0.060	NS
									**24h 48h**	-8	-2.342	0.192	NS

Statistical analysis performed using OriginPRO 2024 v10.1.0.170. The variation in the sample number (*n*) can be attributed to the loss of some samples during the process of freeze-drying or to a variation in the replicate injections that were performed twice. The statistical significance was represented as * ($p < 0.05$) and NS when it is not significant.

Table S4 Statistical analysis of E4 (urolithin C) concentrations along fermentation time.

Normality test			Non-parametric test						Post-hoc test				
**Shapiro-wilk test**			**Kruskal-wallis test**						**Dunn's Test**				
df	stat	*p*-value		*n*	Mean Rank	Chi-Square	*df*	Prob>Chi-Square		Mean Rank Diff	Z	Prob	Sig
7	0.785	0.029	**12h**	3	2	5.357	2	0.068	**12h 24h**	4.667	1.366	0.999	NS
			**24h**	3	2				**12h 48h**	7.5	1.964	0.495	NS
			**48h**	2	3				**24h 48h**	5	1.464	0.999	NS

Statistical analysis performed using OriginPRO 2024 v10.1.0.170. The variation in the sample number (n) can be attributed to the loss of some samples during the process of freeze-drying or to a variation in the replicate injections that were performed twice. The statistical significance was represented as * ($p < 0.05$) and NS when it is not significant.

Table S5 Statistical analysis of E3 (ellagic acid) and E4 (urolithin C) concentrations at 12h of fermentation.

Normality test			Homogeneity of variance test					Non-parametric test					
**Shapiro-wilk test**			**Levene's test**					**Mann-Whitney Test**					
df	stat	*p*-value		*df*	Mean Square	*F*-value	Prob>*F*		*n*	Mean Rank	U	Z	Asymp.Prob>|U|
4	0.783	0.076	**Model**	1	8.22E-5	-	-	**E3**	2	1.5	0	-1.162	0.245
			**Error**	2	0			**E4**	2	3.5			

E3 – ellagic acid; E4 – urolithin C; Statistical analysis performed using OriginPRO 2024 v10.1.0.170. The variation in the sample number (n) can be attributed to the loss of some samples during the process of freeze-drying or to a variation in the replicate injections that were performed twice. The statistical significance was represented as * ($p < 0.05$) and NS when it is not significant.

Table S6 Statistical analysis of E3 (ellagic acid) and E4 (urolithin C) concentrations at 24h of fermentation.

**Normality test**			**Non-parametric test**					
**Shapiro-wilk test**			**Mann-Whitney Test**					
df	stat	*p*-value		*n*	Mean Rank	U	Z	Asymp.Prob>|U|
5	0.717	0.014	**E3**	3	2	0	-1.443	0.149
			**E4**	2	4.5			

E3 – ellagic acid; E4 – urolithin C; Statistical analysis performed using OriginPRO 2024 v10.1.0.170. The variation in the sample number (n) can be attributed to the loss of some samples during the process of freeze-drying or to a variation in the replicate injections that were performed twice. The statistical significance was represented as * ($p < 0.05$) and NS when it is not significant.

Table S7 Statistical analysis of E1 (urolithin D isomer), E2 (urolithin M5), E3 (ellagic acid), E4 (urolithin C), E5 (urolithin B isomer), E6 (urolithin C isomer), E7 ((iso)urolithin A) concentrations at 48h of fermentation.

**Normality test**			**Non-parametric test**						**Post-hoc test**					**Multiple comparisons**					
**Shapiro-wilk test**			**Kruskal-wallis test**						**Dunn's Test**					**Bonferroni correction**		**Holm Correction**		**BH Correction**	
df	stat	*p*-value		*n*	Mean Rank	Chi-Square	*df*	Prob>Chi-Square		Mean Rank Diff	Z	Prob	Sig	Adj *p*-value	Sig	Adj *p*-value	Sig	Adj *p*-value	Sig
25	0.661	<0.0001	**E3**	3	16	21.073	6	0.002	**E3 E4**	-2.333	-0.388	0.999	NS	0.999	NS	0.999	NS	0.999	NS
			**E4**	3	18.333				**E3 E1**	-8	-1.331	0.999	NS	0.999	NS	0.999	NS	0.999	NS
			**E1**	3	24				**E3 E2**	-1.333	-0.222	0.999	NS	0.999	NS	0.999	NS	0.999	NS
			**E2**	3	17.333				**E3 E5**	9	1.498	0.999	NS	0.999	NS	0.999	NS	0.999	NS
			**E5**	3	7				**E3 E6**	3.6	0.670	0.999	NS	0.999	NS	0.999	NS	0.999	NS
			**E6**	5	12.4				**E3 E7**	13	2.419	0.327	NS	0.999	NS	0.999	NS	0.999	NS
			**E7**	5	3				**E4 E1**	-5.667	-0.943	0.999	NS	0.999	NS	0.999	NS	0.999	NS

		**E4 E2**	1	0.166	0.999	NS	0.999	NS	0.999	NS	0.999	NS
		**E4 E5**	11.333	1.886	0.999	NS	0.999	NS	0.999	NS	0.999	NS
		**E4 E5**	5.9333	1.104	0.999	NS	0.999	NS	0.999	NS	0.999	NS
		**E4 E7**	15.333	2.853	0.091	NS	0.999	NS	0.999	NS	0.999	NS
		**E1 E2**	6.667	1.109	0.999	NS	0.999	NS	0.999	NS	0.999	NS
		**E1 E5**	17	2.829	0.098	NS	0.999	NS	0.999	NS	0.719	NS
		**E1 E6**	11.6	2.158	0.649	NS	0.999	NS	0.999	NS	0.999	NS
		**E1 E7**	21	3.907	0.002	*	0.042	*	0.044	*	0.044	*
		**E2 E5**	10.333	1.720	0.999	NS	0.999	NS	0.999	NS	0.999	NS
		**E2 E6**	4.933	0.918	0.999	NS	0.999	NS	0.999	NS	0.999	NS
		**E2 E7**	14.333	2.667	0.161	NS	0.999	NS	0.999	NS	0.886	NS
		**E5 E6**	-5.4	-1.004	0.999	NS	0.999	NS	0.999	NS	0.999	NS
		**E5 E7**	4	0.744	0.999	NS	0.999	NS	0.999	NS	0.999	NS
		**E6 E7**	9.4	2.019	0.912	NS	0.999	NS	0.999	NS	0.999	NS

BH – Benjamini-Hochberg; E1 – urolithin D isomer; E2 – urolithin M5; E3 – ellagic acid; E4 – urolithin C; E5 – urolithin B isomer; E6 – urolithin C isomer; E7 – (iso)urolithin A; Statistical analysis performed using OriginPRO 2024 v10.1.0.170. Statistical analysis performed using OriginPRO 2024 v10.1.0.170. The variation in the sample number (n) can be attributed to the loss of some samples during the process of freeze-drying or to a variation in the replicate injections that were performed twice. The statistical significance was represented as * ($p < 0.05$) and NS when it is not significant.

Table S8 Statistical analysis of N11 (naringenin) concentrations along fermentation time.

**Normality test**			**Homogeneity of variance test**					**Parametric test**						**Post-hoc test**								
**Shapiro-wilk test**			**Levene's test**					**One way ANOVA**						**Tukey's Test**								
df	Stat	*p*-value		*df*	Mean Square	*F*-value	Prob>*F*		*df*	Sum of Squares	Mean Square	*F*-value	Prob>*F*		Mean Diff	SEM	*t*-value	Prob	*α*	Sig	LCL	UCL
12	0.870	0.066	**Model**	3	3.497E-4	0.310	0.818	**Model**	3	0.298	0.010	18.033	6.419E-4	**6h 0h**	-0.402	0.061	9.366	7.513E-4	0.05	*	-0.596	-0.207
			**Error**	8	0.001			**Error**	8	0.044	0.006			**12h 0h**	-0.361	0.061	8.411	0.002	0.05	*	-0.555	-0.167
								**Tot**	11	0.3				**12h**	0.0	0.0	0.9	0.904	0.05	NS	-0.153	0.235

		**al**	43	**6h**	41	61	55					
				**24h 0h**	-0.303	0.061	7.066	0.005	0.05	*	-0.497	-0.109
				**24h 6h**	0.099	0.061	2.300	0.417	0.05	NS	-0.096	0.293
				**24h 12h**	0.058	0.061	1.345	0.779	0.05	NS	-0.137	0.252

Statistical analysis performed using OriginPRO 2024 v10.1.0.170. The variation in the sample number (n) can be attributed to the loss of some samples during the process of freeze-drying or to a variation in the replicate injections that were performed twice. The statistical significance was represented as * ($p < 0.05$) and NS when it is not significant.

Table S9 Statistical analysis of N1 (hydroxybenzoic acid) and N2 (*p*-coumaric acid isomer) co-eluted concentrations along fermentation time.

**Normality test**			**Homogeneity of variance test**					**Parametric test**						**Post-hoc test**								
**Shapiro-wilk test**			**Levene's test**					One way ANOVA						**Tukey's Test**								
df	Stat	*p*-value		*df*	Mean Square	*F*-value	Prob>*F*		*df*	Sum of squares	Mean Square	*F*-value	Prob>*F*		Mean Diff	SEM	*t*-value	Prob	α	Sig	LCL	UCL
6	0.902	0.383	**Model**	1	0.003	0.260	0.637	**Model**	1	0.044	0.044	1.167	0.341	**6h 12h**	-0.106	0.172	0.872	0.571	0.05	NS	-0.582	0.370
			**Error**	4	0.010			**Error**	4	0.150	0.037											
								**Total**	5	0.193												

Statistical analysis performed using OriginPRO 2024 v10.1.0.170. The variation in the sample number (n) can be attributed to the loss of some samples during the process of freeze-drying or to a variation in the replicate injections that were performed twice. The statistical significance was represented as * ($p < 0.05$) and NS when it is not significant.

Table S10 Statistical analysis of N8 (2-(2-hydroxyphenyl)acetic acid) concentrations along fermentation time.

**Normality test**			**Homogeneity of variance test**					**Parametric test**						**Post hoc test**								
**Shapiro-wilk test**			**Levene's test**					**One way ANOVA**						**Tukey's Test**								
df	Stat	*p*-value		*df*	Mean Square	*F*-value	Prob>*F*		*df*	Sum of squares	Mean Square	*F*-value	Prob>*F*		Mean Diff	SEM	*t*-value	Prob	*α*	Sig	LCL	UCL
6	0.940	0.659	**Model**	1	2.83E-5	1.521	0.285	**Model**	1	0.045	0.045	6.755	0.060	**6h 12h**	0.174	0.067	3.676	0.060	0.05	NS	-0.012	0.360
			**Error**	4	1.86E-6			**Error**	4	0.027	0.007											
								**Total**	5	0.072												

Statistical analysis performed using OriginPRO 2024 v10.1.0.170. The variation in the sample number (n) can be attributed to the loss of some samples during the process of freeze-drying or to a variation in the replicate injections that were performed twice. The statistical significance was represented as * ($p < 0.05$) and NS when it is not significant.

Table S11 Statistical analysis of N10 (3-phenylpropanoic acid) concentrations along fermentation time.

**Normality test**			**Homogeneity of variance test**					**Parametric test**						**Post-hoc test**								
**Shapiro-wilk test**			**Levene's test**					One way ANOVA						**Tukey's Test**								
df	Stat	*p*-value		*df*	Mean Square	*F*-value	Prob>*F*		*df*	Sum of squares	Mean Square	*F*-value	Prob>*F*		Mean Diff	SEM	t-value	Prob	*α*	Sig	LCL	UCL
9	0.852	0.078	**Model**	2	0.007	1.330	0.333	**Model**	2	2.260	1.130	30.391	7.252E-4	**6h 12h**	-0.107	0.157	0.957	0.785	0.05	NS	-0.590	0.377
			**Error**	6	0.006			**Error**	6	0.223	0.037			**6h 24h**	1.008	0.157	9.034	0.002	0.05	*	0.523	1.489
								**Total**	8	2.483				**12h 24h**	1.112	0.157	9.991	9.831E-4	0.05	*	0.629	1.595

Statistical analysis performed using OriginPRO 2024 v10.1.0.170. The variation in the sample number (n) can be attributed to the loss of some samples during the process of freeze-drying or to a variation in the replicate injections that were performed twice. The statistical significance was represented as * ($p < 0.05$) and NS when it is not significant.

Table S12 Statistical analysis of N11 (naringenin), N1 + N2 (hydroxybenzoic acid and p-coumaric acid isomer co-eluted), N8 (2-(2-hydroxyphenyl)acetic acid), N10 (3-phenylpropanoic acid) and N9 (3-(hydroxyphenyl)propanoic acid) concentrations at 6h fermentation.

**Normality test**			**Homogeneity of variance test**					**Parametric test**						**Post-hoc test**								
**Shapiro-wilk test**			**Levene's test**					**One way ANOVA**						**Tukey's Test**								
df	Stat	*p*-value		*df*	Mean Square	*F*-value	Prob>*F*		*df*	Sum of squares	Mean Square	*F*-value	Prob>*F*		Mean Diff	SEM	t-value	Prob	*α*	Sig	LCL	UCL
15	0.944	0.432	**Model**	4	0.017	3.243	0.060	**Model**	4	1.058	0.265	8.098	0.004	**N1+N2 N11**	-0.030	0.148	0.287	0.100	0.05	NS	-0.516	0.456
			**Error**	10	0.005			**Error**	10	0.327	0.033			**N8 N11**	-0.198	0.148	1.894	0.675	0.05	NS	-0.683	0.288
								**Total**	14	1.385				**N8 N1+N2**	-0.168	0.148	1.607	0.785	0.05	NS	-0.653	0.318
														**N10 N11**	0.579	0.148	5.553	0.019	0.05	*	0.094	1.065
														**N10 N1+N2**	0.609	0.148	5.841	0.014	0.05	*	0.124	1.095
														**N10 N8**	0.777	0.148	7.447	0.003	0.05	*	0.292	1.263
														**N9 N11**	0.187	0.148	1.795	0.714	0.05	NS	-0.298	0.673
														**N9 N1+N2**	0.217	0.148	2.082	0.600	0.05	NS	-0.268	0.703
														**N9 N8**	0.385	0.148	3.689	0.142	0.05	NS	-0.101	0.871
														**N9 N10**	-0.392	0.148	3.758	0.132	0.05	NS	-0.878	0.094

N11 – naringenin; N1 + N2 – hydroxybenzoic acid and p-coumaric acid isomer co-eluted; N8 – 2-(2-hydroxyphenyl)acetic acid; N10 – 3-phenylpropanoic acid; N9 – 3-(hydroxyphenyl)propanoic acid; Statistical analysis performed using OriginPRO 2024 v10.1.0.170. The variation in the sample number (n) can be attributed to the loss of some samples during the process of freeze-drying or to a variation in the replicate injections that were performed twice. The statistical significance was represented as * ($p < 0.05$) and NS when it is not significant.

Table S13 Statistical analysis of N11 (naringenin), N1+N2 (hydroxybenzoic acid and p-coumaric acid isomer co-eluted), N8 (2-(2-hydroxyphenyl)acetic acid, N10 (3-phenylpropanoic acid) and N6 (3-(2,4-dihydroxyphenyl)propanoic acid)) concentrations at 12h fermentation.

**Normality test**			**Homogeneity of variance test**					**Parametric test**						**Post-Hoc test**								
**Shapiro-wilk test**			**Levene's test**					**One way ANOVA**						**Tukey's Test**								
df	Stat	*p*-value		*df*	Mean Square	*F*-value	Prob>*F*		*df*	Sum of squares	Mean Square	*F*-value	Prob>*F*		Mean Diff	SEM	t-value	Prob	*α*	Sig	LCL	UCL
15	0.913	0.149	**Model**	4	0.003	1.041	0.433	**Model**	4	0.639	0.160	11.195	0.001	**N1+N2 N11**	-0.177	0.098	2.561	0.419	0.05	NS	-0.498	0.144
			**Error**	10	0.003			**Error**	10	0.143	0.014			**N8 N11**	-0.065	0.098	0.937	0.960	0.05	NS	-0.386	0.256
								**Total**	14	0.782				**N8 N1+N2**	0.112	0.098	1.623	0.779	0.05	NS	-0.209	0.433
														**N10 N11**	0.432	0.098	6.264	0.009	0.05	*	0.111	0.753
														**N10 N1+N2**	0.609	0.098	8.824	7.098E-4	0.05	*	0.288	0.930
														**N10 N8**	0.497	0.098	7.201	0.003	0.05	*	0.176	0.818
														**N6 N11**	0.035	0.098	0.510	0.996	0.05	NS	-0.286	0.356
														**N6 N1+N2**	0.212	0.098	3.071	0.265	0.05	NS	-0.109	0.533
														**N6 N8**	0.010	0.098	1.447	0.839	0.05	NS	-0.221	0.421
														**N6 N10**	-0.397	0.098	5.754	0.015	0.05	*	-0.718	-0.076

N11 – naringenin; N1 + N2 – hydroxybenzoic acid and p-coumaric acid isomer co-eluted; N8 – 2-(2-hydroxyphenyl)acetic acid; N10 – 3-phenylpropanoic acid; N6 – 3-(2,4-dihydroxyphenyl)propanoic acid; Statistical analysis performed using OriginPRO 2024 v10.1.0.170. The variation in the sample number (n) can be attributed to the loss of some samples during the process of freeze-drying or to a variation in the replicate injections that were performed twice. The statistical significance was represented as * ($p < 0.05$) and NS when it is not significant.

Table S14 Statistical analysis of N11 (naringenin), N10 (3-phenylpropanoic acid) and N7 (2-(4-hydroxyphenyl)acetic acid) concentrations at 24h fermentation.

**Normality test**			**Non-parametric test**						**Post-hoc test**					**Multiple comparisons**					
**Shapiro-wilk test**			**Kruskal-wallis test**						**Dunn's Test**					**Bonferroni correction**		**Holm correction**		**BH correction**	
df	stat	*p*-value		*n*	Mean Rank	Chi-Square	*df*	Prob>Chi-Square		Mean Rank Diff	Z	Prob	Sig	Adj *p*-value	Sig	Adj *p*-value	Sig	Adj *p*-value	Sig
9	0.769	0.009	**N11**	3	4.333	5.956	2	0.051	**N11 N10**	-3.667	-1.640	0.303	NS	0.909	NS	0.606	NS	0.455	NS
			**N10**	3	8				**N11 N7**	1.667	0.745	0.999	NS	0.999	NS	0.999	NS	0.999	NS
			**N7**	3	2.667				**N10 N7**	5.333	2.385	0.051	NS	0.153	NS	0.153	NS	0.153	NS

N11 – naringenin; N10 – 3-phenylpropanoic acid; N7 – 2-(4-hydroxyphenyl)acetic acid; Statistical analysis performed using OriginPRO 2024 v10.1.0.170. The variation in the sample number (n) can be attributed to the loss of some samples during the process of freeze-drying or to a variation in the replicate injections that were performed twice. The statistical significance was represented as * ($p < 0.05$) and NS when it is not significant.

Table S15 Statistical analysis of P1 (phloroglucinol) concentrations along fermentation time.

**Normality test**			**Homogeneity of variance test**					**Non-parametric test**						**Post-hoc test**				
**Shapiro-wilk test**			**Levene's test**					**Kruskal-wallis test**						**Dunn's Test**				
df	stat	*p*-value		*df*	Mean Square	*F*-value	Prob>*F*		*n*	Mean Rank	Chi-Square	*df*	Prob>Chi-Square		Mean Rank Diff	Z	Prob	Sig
24	0.942	0.181	**Model**	4	4735.4	4.400	0.012	**0h**	4	18.75	8.841	4	0.065	**0h 6h**	10.75	2.266	0.234	NS
			**Error**	19	1076.2			**6h**	5	8				**0h 12h**	3.55	0.748	0.999	NS
								**12h**	5	15.2				**0h 24h**	11.35	2.393	0.167	NS
								**24h**	5	7.4				**0h 48h**	4.35	0.917	0.999	NS
								**48h**	5	14.4				**6h 12h**	-7.2	-1.610	0.999	NS
														**6h 24h**	0.6	0.134	0.999	NS
														**6h 48h**	-6.4	-1.431	0.999	NS
														**12h 24h**	7.8	1.744	0.811	NS
														**12h 48h**	0.8	0.179	0.999	NS
														**24h 48h**	-7	-1.565	0.999	NS

Statistical analysis performed using OriginPRO 2024 v10.1.0.170. The variation in the sample number (n) can be attributed to the loss of some samples during the process of freeze-drying or to a variation in the replicate injections that were performed twice. The statistical significance was represented as * ($p < 0.05$) and NS when it is not significant.

Table S16 Statistical analysis of P2 (phloroglucinic acid) concentrations along fermentation time.

**Normality test**			**Homogeneity of variance test**					**Non-parametric test**						**Post-hoc test**				
**Shapiro-wilk test**			**Levene's test**					**Kruskal-wallis test**						**Dunn's Test**				
df	stat	*p*-value		*df*	Mean Square	*F*-value	Prob>*F*		*n*	Mean Rank	Chi-Square	*df*	Prob>Chi-Square		Mean Rank Diff	Z	Prob	Sig
16	0.930	0.243	**Model**	3	0.040	15.003	2.31E-4	**6h**	3	8.333	1.141	3	0.767	**6h 12h**	1.333	0.384	0.999	NS
			**Error**	12	0.003			**12h**	5	7				**6h 24h**	-0.467	-0.134	0.999	NS
								**24h**	5	8.8				**6h 48h**	-2.333	-0.600	0.999	NS
								**48h**	3	10.667				**12h 24h**	-1.799	-0.598	0.999	NS
														**12h 48h**	-3.667	-1.055	0.999	NS
														**24h 48h**	-1.867	-0.537	0.999	NS

Statistical analysis performed using OriginPRO 2024 v10.1.0.170. The variation in the sample number (*n*) can be attributed to the loss of some samples during the process of freeze-drying or to a variation in the replicate injections that were performed twice. The statistical significance was represented as * ($p < 0.05$) and NS when it is not significant.

Table S17 Statistical analysis of P3 (2-hydroxy-3-phenylpropanoic acid) concentrations along fermentation time.

**Normality test**			**Homogeneity of variance test**					**Parametric test**						**Post-hoc test**								
**Shapiro-wilk test**			**Levene's test**					**One way ANOVA**						**Tukey's Test**								
df	Stat	*p*-value		*df*	Mean Square	*F*-value	Prob>*F*		*df*	Sum of squares	Mean Square	*F*-value	Prob>*F*		Mean Diff	SEM	t-value	Prob	*α*	Sig	LCL	UCL
15	0.906	0.118	**Model**	3	0.108	1.228	0.346	**Model**	3	12.978	4.326	9.341	0.002	**12h 6h**	-0.355	0.481	1.045	0.879	0.05	NS	-1.804	1.093
			**Error**	11	0.088			**Error**	11	5.094	0.463			**24h 6h**	0.726	0.520	1.975	0.526	0.05	NS	-0.838	2.290
								**Total**	14	18.072				**24h 12h**	1.082	0.520	2.943	0.218	0.05	NS	-0.483	2.646
														**48h 6h**	2.000	0.481	5.878	0.007	0.05	*	0.552	3.448
														**48h 12h**	2.356	0.481	6.923	0.002	0.05	*	0.9075	3.804
														**48h 24h**	1.274	0.520	3.467	0.124	0.05	NS	-0.290	2.839

Statistical analysis performed using OriginPRO 2024 v10.1.0.170. The variation in the sample number (n) can be attributed to the loss of some samples during the process of freeze-drying or to a variation in the replicate injections that were performed twice. The statistical significance was represented as * ($p < 0.05$) and NS when it is not significant.

Table S18 Statistical analysis of P1 (phloroglucinol), P2 (phloroglucinic acid), P3 (2-hydroxy-3-phenylpropanoic acid) concentrations at 6h fermentation.

**Normality test**			**Non-parametric test**						**Post-hoc test**					**Multiple comparisons**					
**Shapiro-wilk test**			**Kruskal-wallis test**						**Dunn's Test**					**Bonferroni correction**		**Holm correction**		**BH correction**	
df	stat	*p*-value		*n*	Mean Rank	Chi-Square	*df*	Prob>Chi-Square		Mean Rank Diff	Z	Prob	Sig	Adj *p*-value	Sig	Adj *p*-value	Sig	Adj *p*-value	Sig
12	0.729	0.002	**P1**	5	10	9.692	2	0.008	**P1 P2**	8	3.038	0.007	*	0.021	*	0.021	*	0.021	*
			**P2**	3	2				**P1 P3**	4.5	1.861	0.188	NS	0.564	NS	0.376	NS	0.282	NS
			**P3**	4	5.5				**P2 P3**	-3.5	-1.271	0.611	NS	0.999	NS	0.611	NS	0.611	NS

P1 – Phloroglucinol; P2 – Phloroglucinic acid; P3 – 2-hydroxy-3-phenylpropanoic acid; The variation in the sample number (n) can be attributed to the loss of some samples during the process of freeze-drying or to a variation in the replicate injections that were performed twice. The statistical significance was represented as * ($p < 0.05$) and NS when it is not significant.

Table S19 Statistical analysis of P1 (phloroglucinol), P2 (Phloroglucinic acid), P3 (2-hydroxy-3-phenylpropanoic acid) concentrations at 12h fermentation.

**Normality test**			**Non-parametric test**						**Post-hoc test**					**Multiple comparisons**					
**Shapiro-wilk test**			**Kruskal-wallis test**						**Dunn's Test**					**Bonferroni correction**		**Holm correction**		**BH correction**	
df	stat	*p*-value		*n*	Mean Rank	Chi-Square	*df*	Prob>Chi-Square		Mean Rank Diff	Z	Prob	Sig	Adj *p*-value	Sig	Adj *p*-value	Sig	Adj *p*-value	Sig
14	0.672	1.93E-4	**P1**	5	12	11.571	2	0.003	**P1 P2**	9	3.402	0.002	*	0.006	*	0.006	*	0.006	*
			**P2**	5	3				**P1 P3**	4.5	1.604	0.326	NS	0.978	NS	0.652	NS	0.489	NS
			**P3**	4	7.5				**P2 P3**	-4.5	-1.604	0.326	NS	0.978	NS	0.652	NS	0.489	NS

P1 – Phloroglucinol; P2 – Phloroglucinic acid; P3 – 2-hydroxy-3-phenylpropanoic acid; Statistical analysis performed using OriginPRO 2024 v10.1.0.170. The variation in the sample number (n) can be attributed to the loss of some samples during the process of freeze-drying or to a variation in the replicate injections that were performed twice. The statistical significance was represented as * ($p < 0.05$) and NS when it is not significant.

Table S20 Statistical analysis of P1 (phloroglucinol), P2 (phloroglucinic acid), P3 (2-hydroxy-3-phenylpropanoic acid) concentrations at 24h fermentation.

**Normality test**			**Non-parametric test**						**Post-hoc test**					**Multiple comparisons**					
**Shapiro-wilk test**			**Kruskal-wallis test**						**Dunn's Test**					**Bonferroni correction**		**Holm correction**		**BH correction**	
df	stat	*p*-value		*n*	Mean Rank	Chi-Square	*df*	Prob>Chi-Square		Mean Rank Diff	Z	Prob	Sig	Adj *p*-value	Sig	Adj *p*-value	Sig	Adj *p*-value	Sig
13	0.639	1.44E-4	**P1**	5	11	10.550	2	0.005	**P1 P2**	8	3.248	0.004	*	0.012	*	0.012	*	0.012	*
			**P2**	5	3				**P1 P3**	4	1.406	0.479	NS	0.999	NS	0.958	NS	0.719	NS
			**P3**	3	7				**P2 P3**	-4	-1.406	0.479	NS	0.999	NS	0.958	NS	0.719	NS

P1 – Phloroglucinol; P2 – Phloroglucinic acid; P3 – 2-hydroxy-3-phenylpropanoic acid; Statistical analysis performed using OriginPRO 2024 v10.1.0.170. The variation in the sample number (n) can be attributed to the loss of some samples during the process of freeze-drying or to a variation in the replicate injections that were performed twice. The statistical significance was represented as * ($p < 0.05$) and NS when it is not significant.

Table S21 Statistical analysis of P1 (phloroglucinol), P2 (phloroglucinic acid), P3 (2-hydroxy-3-phenylpropanoic acid) concentrations at 48h fermentation.

**Normality test**			**Non-parametric test**						**Post-hoc test**					**Multiple comparisons**					
**Shapiro-wilk test**			**Kruskal-wallis test**						**Dunn's Test**					**Bonferroni correction**		**Holm correction**		**BH correction**	
df	stat	*p*-value		*n*	Mean Rank	Chi-Square	*df*	Prob>Chi-Square		Mean Rank Diff	Z	Prob	Sig	Adj *p*-value	Sig	Adj *p*-value	Sig	Adj *p*-value	Sig
12	0.668	4.25E-4	**P1**	5	10	9.692	2	0.008	**P1 P2**	8	3.038	0.007	*	0.021	*	0.021	*	0.021	*
			**P2**	3	2				**P1 P3**	4.5	1.861	0.188	NS	0.564	NS	0.376	NS	0.282	NS
			**P3**	4	5.5				**P2 P3**	-3.5	-1.271	0.611	NS	0.999	NS	0.611	NS	0.611	NS

P1 – Phloroglucinol; P2 – Phloroglucinic acid; P3 – 2-hydroxy-3-phenylpropanoic acid; Statistical analysis performed using OriginPRO 2024 v10.1.0.170. The variation in the sample number (n) can be attributed to the loss of some samples during the process of freeze-drying or to a variation in the replicate injections that were performed twice. The statistical significance was represented as * ($p < 0.05$) and NS when it is not significant.

References

1. Tu J, Li Q, Zhou B (2021) The tannins from *Sanguisorba officinalis* l. (Rosaceae): A systematic study on the metabolites of rats based on HPLC–LTQ–Orbitrap MS^2 analysis. Molecules 26:. https://doi.org/10.3390/molecules26134053

2. García-Villalba R, Espín JC, Tomás-Barberán FA (2016) Chromatographic and spectroscopic characterization of urolithins for their determination in biological samples after the intake of foods containing ellagitannins and ellagic acid. J Chromatogr A 1428:162–175. https://doi.org/10.1016/j.chroma.2015.08.044

3. Wu S, Li H, Li G, et al (2020) Metabolite identification of gut microflora-cassia seed interactions using UPLC-QTOF/MS. Exp Ther Med. https://doi.org/10.3892/etm.2020.8585

4. Tomás-Barberán FA, González-Sarrías A, García-Villalba R, et al (2017) Urolithins, the rescue of “old” metabolites to understand a “new” concept: Metabotypes as a nexus among phenolic metabolism, microbiota dysbiosis, and host health status. Mol Nutr Food Res 61:1500901. https://doi.org/10.1002/mnfr.201500901

5. Bayle M, Roques C, Marion B, et al (2016) Development and validation of a liquid chromatography-electrospray ionization-tandem mass spectrometry method for the determination of urolithin C in rat plasma and its application to a pharmacokinetic study. J Pharm Biomed Anal 131:33–39. https://doi.org/10.1016/j.jpba.2016.07.046

6. Lech K, Fornal E (2020) A mass spectrometry-based approach for characterization of red, blue, and purple natural dyes. Molecules 2020, Vol 25, Page 3223 25:3223. https://doi.org/10.3390/MOLECULES25143223

7. Laveriano-Santos EP, Marhuenda-Muñoz M, Vallverdú-Queralt A, et al (2022) Identification and quantification of urinary microbial phenolic metabolites by HPLC-ESI-LTQ-orbitrap-HRMS and their relationship with dietary polyphenols in adolescents. Antioxidants 11:1167. https://doi.org/10.3390/antiox11061167

8. Santos SAO, Vilela C, Freire CSR, et al (2013) Ultra-high performance liquid chromatography coupled to mass spectrometry applied to the identification of valuable phenolic compounds from *Eucalyptus* wood. J Chromatogr B Analyt Technol Biomed Life Sci 938:65–74. https://doi.org/10.1016/j.jchromb.2013.08.034

9. Martínez-Huélamo M, Tulipani S, Jáuregui O, et al (2015) Sensitive and rapid UHPLC-MS/MS for the analysis of tomato phenolics in human biological samples. Molecules 20:20409–20425. https://doi.org/10.3390/molecules201119702

10. Ibdah M, Gang DR (2014) Use of coupled ion mobility spectrometry-time of flight mass spectrometry to analyze saturated and unsaturated phenylpropanoic acids and chalcones. Chem Cent J 8:1–9. https://doi.org/10.1186/1752-153X-8-38

11. Zhang B, Zhang Y, Liu X, et al (2023) Distinctive anti-inflammatory effects of resveratrol, dihydroresveratrol, and 3-(4-hydroxyphenyl)-propionic acid on DSS-induced colitis in pseudo-germ-free mice. Food Chem 400:133904. https://doi.org/10.1016/j.foodchem.2022.133904
